# RNA dysregulation in neurodegenerative diseases

**DOI:** 10.1038/s44318-024-00352-6

**Published:** 2025-01-09

**Authors:** Yini Li, Shuying Sun

**Affiliations:** 1 Department of Physiology, Johns Hopkins University School of Medicine, Baltimore, MD 21205 USA; 2 Brain Science Institute, Johns Hopkins University School of Medicine, Baltimore, MD 21205 USA; 3 Departments of Neuroscience, Pathology, Johns Hopkins University School of Medicine, Baltimore, MD 21205 USA

**Keywords:** Neurodegenerative Diseases, RNA Metabolism, RNA-binding Protein, RNA-targeting Therapeutics, Neuroscience, Pharmacology & Drug Discovery, RNA Biology

## Abstract

Dysregulation of RNA processing has in recent years emerged as a significant contributor to neurodegeneration. The diverse mechanisms and molecular functions underlying RNA processing underscore the essential role of RNA regulation in maintaining neuronal health and function. RNA molecules are bound by RNA-binding proteins (RBPs), and interactions between RNAs and RBPs are commonly affected in neurodegeneration. In this review, we highlight recent progress in understanding dysregulated RNA-processing pathways and the causes of RBP dysfunction across various neurodegenerative diseases. We discuss both established and emerging mechanisms of RNA-mediated neuropathogenesis in this rapidly evolving field. Furthermore, we explore the development of potential RNA-targeting therapeutic approaches for the treatment of neurodegenerative diseases.

## Abstract (web summary)

This review discusses how aberrant RNA processing contributes to neurodegeneration and can be targeted for the development of efficient therapeutic strategies.

## Graphical abstract

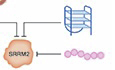

## Introduction

Neurodegenerative diseases are prevalent age-related diseases, with Alzheimer’s disease (AD) as a prototype of such conditions. As of 2024, approximately 6.9 million Americans are affected by AD, making it the most common neurodegenerative disease, followed by Parkinson’s disease (PD) (from the Alzheimer’s Disease Association Report (2024)). There are also many less prevalent or rare neurodegenerative diseases such as Huntington’s disease (HD), frontotemporal dementia (FTD) and amyotrophic lateral sclerosis (ALS). With the global demographic trend of population aging, it is predicted that an increasing number of people will be diagnosed with neurodegenerative diseases globally (Collaborators, 2024). Though the clinical symptoms of these diseases vary, multiple neurodegenerative diseases share similar underlying pathological mechanisms (Abramzon et al, 2020; Jellinger, 2010; Ling et al, 2013; Paulson, 2018; Wingo et al, 2022). The presence of pathological inclusions and causative mutations of RNA-binding proteins (RBPs) is increasingly observed among neurodegenerative diseases. In addition, pathological repeat expansion in multiple diseases, such as ALS, FTD, HD and various types of spinocerebellar ataxia, yields repeat-containing RNAs that could cause neurotoxicity via various mechanisms (Paulson, 2018). In the post-genomic era, a variety of RNA processing pathways and emerging types of coding and noncoding RNAs have been commonly identified in the disease context (Statello et al, 2021), with potential contributions to neurodegeneration. Therapeutic strategies targeting RNA to modulate disease-linked genes have achieved significant success (Khorkova et al, 2023; Zhu et al, 2022).

**Figure 1:**
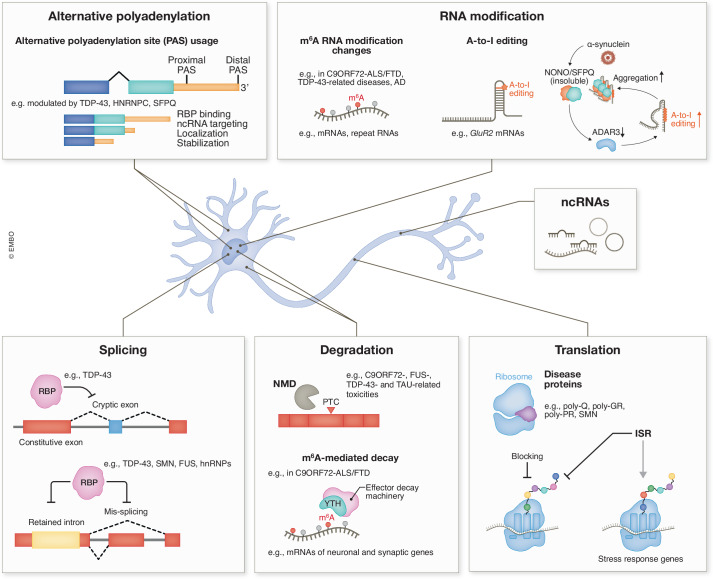
Dysregulation of RNA processing in neurodegenerative diseases. RNA dysregulation can occur in almost all steps of RNA processing, including RNA modification, splicing, polyadenylation, degradation, and translation. The dysregulated RNAs can be protein-coding mRNAs or noncoding RNAs (ncRNAs).

**Figure 2:**
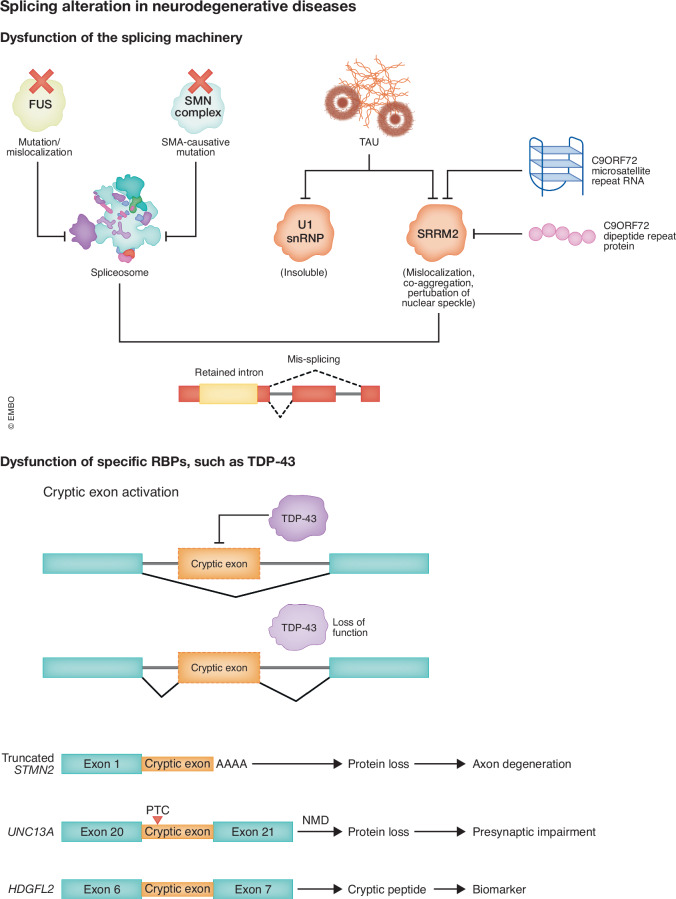
Examples of splicing alterations in different disease contexts. Disease-related splicing changes can be caused by defects in the core splicing machinery, or by dysfunction of specific RNA-binding proteins, such as TDP-43.

**Figure 3:**
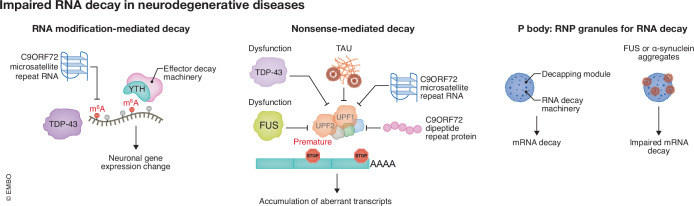
Impaired RNA decay mechanisms in different disease contexts. RNA decay can be disrupted via dysregulated RNA modifications, perturbed nonsense-mediated decay pathways, and dysfunction of decay-related RNP granules, such as P-bodies.

**Figure 4:**
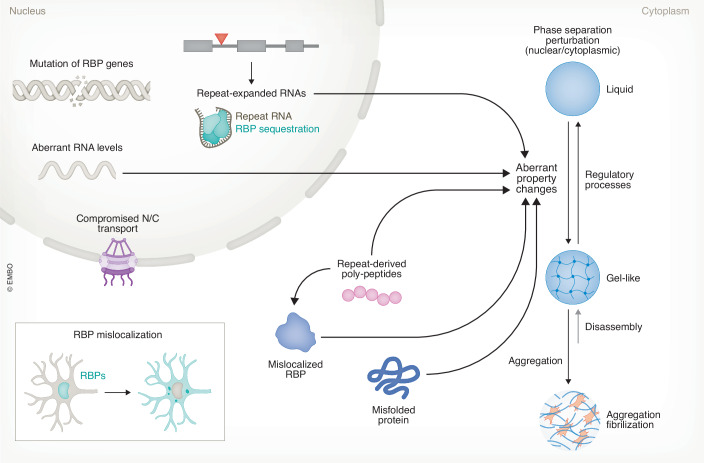
Potential causes of RNA dysregulation in neurodegenerative diseases. RNA dysregulation is primarily driven by RBP dysfunction, which can be attributed to genetic mutations, abnormal expression levels, mislocalization, aberrant phase separation or aggregation, or perturbation by repeat expansions. Many RBPs are multifunctional and can impact a wide range of RNA processing pathways.

**Figure 5:**
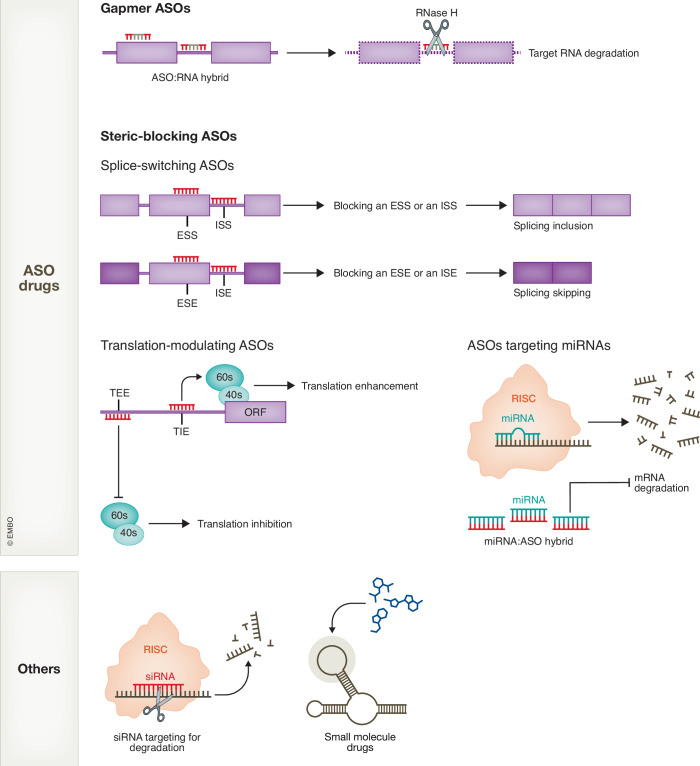
Overview of RNA-targeting therapeutic approaches. Antisense oligonucleotide (ASO) drugs can be classified according to their modes of action. Gapmer ASOs bind to target mRNAs, recruiting endogenous RNase H for target mRNA degradation. Steric-blocking ASOs bind to target mRNAs without inducing degradation; instead, they modulate RNA processing, such as splicing and translation, by preventing RBP binding or altering the RNA structure. They can also base-pair with miRNAs to prevent them from targeting mRNAs for decay. ESS/ISS exonic/intronic splicing-suppressor element, ESE/ISE exonic/intronic splicing-enhancer element, TEE translation-enhancing element, TIE translation-inhibitory element. In addition to ASO drugs, other options for RNA-targeting therapies include siRNAs, which promote mRNA decay, and small-molecule drugs that target specific RNA structures to modulate their processing.

Here, we focus on RNA-related pathogenic mechanisms in neurodegenerative diseases and updates on RNA-targeting therapeutic approaches that hold great promise. We review recent discoveries alongside previous key findings, aiming to offer a timely reference for research on RNA and neurodegenerative diseases, with a particular emphasis on ALS. We start with the various RNA processing pathways and provide representative examples of how these pathways are dysregulated in neurodegenerative diseases (Figs. 1–3). Next, we discuss the mechanisms that lead to RBP dysfunction (Fig. 4), resulting in dysregulation of RNA processing. Finally, we review the current progress in RNA-targeting therapeutics (Fig. 5). The different RNA processing pathways are often interconnected, and most RBPs have multifunctional roles across several RNA processing steps, creating significant interplay among them. Overall, these findings highlight RNA metabolism as a critical factor in disease mechanisms.

### RNA splicing

RNA splicing is a key regulatory step in gene expression during the RNA life cycle. In this process, introns are removed from the nascent pre-messenger RNA (pre-mRNA) and exons are joined to produce the fully processed mRNA. The vast majority of human genes (92–94%) undergo alternative splicing (Wang et al, 2008). Alternative splicing is disproportionally abundant and evolutionarily conserved in the brain (Barbosa-Morais et al, 2012), especially enriched in genes associated with highly specialized neuronal functions, such as synaptic transmission, axon guidance, actin cytoskeleton reorganization, and plasticity (Barbosa-Morais et al, 2012; Merkin et al, 2012; Wang et al, 2008).

Splicing dysregulation can be directly triggered by perturbed RBP function and altered RBP-spliceosome interaction due to disease-related mutations or pathologies. One example is TDP-43, related proteinopathies of which (i.e., nuclear clearance and cytosolic inclusion) are broadly found in about 97% of ALS, 50% of FTD, and 40-60% of AD patients (Ayala et al, 2008; Jo et al, 2020; Meneses et al, 2021). While mutations in TDP-43 account for 5% of familial ALS cases, TDP-43 pathology is also observed in sporadic disease. Increasing evidence suggests that the loss of nuclear TDP-43 is likely an early pathologic event preceding cytosolic aggregation (Sun et al, 2017; Vatsavayai et al, 2016). The function of TDP-43 on alternative splicing has been extensively investigated. Widespread splicing changes have been reported in TDP-43 loss-of-function in vitro and in vivo models (Polymenidou et al, 2011; Tollervey et al, 2011), and in models expressing causative TDP-43 mutations, such as TDP-43^Q331K^ (Arnold et al, 2013) and TDP-43^M337V^ (Watanabe et al, 2020).

Recently, TDP-43-regulated cryptic exons have widely attracted attention. Cryptic exons (CEs) represent a type of alternative splicing in which non-conserved intronic sequences are erroneously included in mature RNAs. Normally, binding of TDP-43 to intronic (UG)n-rich sequences suppresses the recognition of cryptic splice sites. With nuclear clearance of TDP-43, these splice sites are de-repressed, leading to the inclusion of cryptic exons in the mRNA (Fig. 2). Currently, about 100 CE-containing transcripts have been identified in TDP-43-deficient cells. Most identified TDP-43-mediated CEs cause frameshifts and introduce premature stop codons, while there are also some CEs linked to alternative transcription start sites, premature polyadenylation sites, and expansion of conserved exons of the mRNAs (Ling et al, 2015). Consequently, those transcripts containing splicing errors are often the targets of nonsense-mediated decay (NMD) or other surveillance RNA decay pathways. CEs in *STMN2 and UNC13A* are two examples that have been characterized in detail. Stathmin2 (*STMN2)* is a microtubule-related gene. The CE inclusion of *STMN2* exposes cryptic premature polyadenylation sites and reduces the levels of the functional full-length protein (Klim et al, 2019; Krus et al, 2022; Melamed et al, 2019; San Juan et al, 2022). Constitutive *Stmn2* knockout in mice results in delayed microtubule polymerization and axon outgrowth (Krus et al, 2022), and persistent loss of stathmin-2 in adult mice results in ALS-linked pathologies, including reduced inter-neurofilament spacing, axonal caliber collapse, progressive motor and sensory deficits, and muscle denervation (Lopez-Erauskin et al, 2024; San Juan et al, 2022). Another example is *UNC13A*, a gene encoding a presynaptic protein. Loss of *UNC13A* leads to synaptic defects in mice and uncoordinated movement behavior in *Caenorhabditis elegans* (Augustin et al, 1999; Sulston and Brenner, 1974). The out-of-frame CE inclusion results in transcripts degraded by NMD and decreases the functional protein level (Brown et al, 2022; Ma et al, 2022). In addition, TDP-43 pathology is also commonly observed in AD (Meneses et al, 2021). As expected, a high frequency of CE inclusion was detected in a large cohort of AD brains (Hsieh et al, 2019; Sun et al, 2017). The CE of *STMN2* and *UNC13A* have been detected in TDP-43-associated AD (Agra Almeida Quadros et al, 2024). The study within the Religious Orders Study and Rush Memory and Aging Project (ROSMAP) also found that CE events concomitantly increase with Tau pathological burden (Hsieh et al, 2019).

On the other hand, isoforms with in-frame cryptic exons could lead to the production of proteins containing cryptic peptides in diseased neurons. These could be used as biomarkers for monitoring TDP-43 loss of function and disease stage. *HDGFL2* CE and its cryptic peptide is a good example. Elevated levels of HDGFL2 cryptic peptide can be detected in fluid samples from ALS/FTD patients, including those that carry disease-causative mutations but remain asymptomatic (Calliari et al, 2024; Irwin et al, 2024; Seddighi et al, 2024), showing great potential as a biomarker for ALS and FTD. Moreover, an increase in HDGFL2 levels appears to follow a pattern similar to that of the established biomarker neurofilament light chain (NfL), with evidence indicating that it may appear even earlier than the NfL marker (Irwin et al, 2024). A larger clinical enrollment to validate the reliability and sensitivity of its usage as a clinical biomarker is anticipated.

Besides mis-splicing caused by specific RBPs, defects of the general splicing machinery are also observed in neurodegenerative diseases (Fig. 2). Deficiency of the survival motor neuron (SMN) protein by genetic lesions in the *SMN1* gene is the cause of spinal muscular atrophy (SMA), a motor neuron degenerative disease (Lefebvre et al, 1995). SMN deficiency compromises the assembly of small nuclear ribonucleoproteins (snRNPs), the core components of the spliceosome (Battle et al, 2006), thereby resulting in widespread splicing defects in SMA (Lotti et al, 2012; Zhang et al, 2008). Additionally, FUS has been shown to interact with SMN, linking splicing defects in ALS and SMA (Mirra et al, 2017; Sun et al, 2015a; Yamazaki et al, 2012). In ALS caused by mutations in FUS/TLS (FUS-ALS), the cytosolic mislocalization of FUS disrupts the normal interaction between U1 snRNP and SMN complexes, which leads to perturbed snRNP assembly and RNA splicing in FUS-ALS (Jutzi et al, 2020; Sun et al, 2015a). Increased intron retention in many RBPs is observed in FUS mutant cells, which includes *FUS* mRNA itself (Humphrey et al, 2020; Luisier et al, 2018), forming a positive feedback loop in enhancing the splicing defects in FUS-ALS.

RNA splicing alterations also occur in AD. One identified reason is that U1 snRNP spliceosome components accumulate in the insoluble fraction, which perturbs the functionality of the spliceosome (Bai et al, 2013). In particular, the core subunit U1-70K was found to be cleaved to a N-terminal 40-KDa fragment (N40K), which exhibits a dominant-negative effect inhibiting the U1-70K function. This leads to mis-splicing and reduced expression of GABAergic synaptic genes, contributing to hyperexcitability in AD (Chen et al, 2022b). Furthermore, Tau aggregates were recently shown to induce cytoplasmic mislocalization and co-aggregation of nuclear speckle proteins, such as SRRM2 (Lester et al, 2021). Nuclear speckles are membraneless RNP granules enriched in the components of the RNA splicing machinery (Spector and Lamond, 2011). The cytosolic mislocalization leads to global splicing deficits, particularly increased intron retention. Alternative splicing can potentially impact various signaling pathways and thereby contribute to the molecular and cellular phenotypes of AD, including synaptic dysregulation, neuronal hyperexcitability, neuroinflammation, and chromosomal instability (Chen et al, 2022b; Li et al, 2021). Overall, multiple molecular mechanisms underly the RNA splicing dysregulation in AD patients. Further elucidation of how each distinct pathway contributes to disease pathogenesis and how these different mechanisms influence each other requires additional studies.

Deficiency of spliceosomal function is also found in microsatellite repeat expansion diseases. The poly-dipeptide proteins derived from the *C9ORF72* microsatellite repeat expansion are able to block spliceosomal assembly by interacting with U2 snRNP (Yin et al, 2017). Furthermore, it was recently identified that the (GGGGCC)n repeat RNA co-localizes with nuclear speckles and affects its dynamic properties, and the poly-GR can induce SRRM2 cytoplasmic mislocalization and co-aggregation. The repeat RNA and dipeptide proteins synergistically lead to nuclear speckle dysfunction and global splicing deficits, most notably increased exon skipping and intron retention (Wu et al, 2024).

In summary, global RNA splicing dysregulation is broadly found in neurodegenerative diseases (Figs. 1 and 2), although the molecular mechanisms and the exact targets differ. In this review, we focused on examples highlighting that the perturbation of specific RBPs or spliceosomal machinery can trigger splicing defects, as this is more likely to be directly associated with pathogenic mechanisms for disease initiation and progression. The toxicity of RBPs (such as TDP-43 and FUS) usually includes both loss of function, due to nuclear clearance, and gain of toxicity arising from protein aggregation in the cytoplasm. Elucidating the distinct contributions to neurodegeneration is critical for designing effective therapeutic strategies. For TDP-43, increasing evidence suggests that nuclear clearance occurs before aggregation, indicating the significant contribution of splicing dysregulation in driving disease progression. The dysfunction of RBPs and the spliceosome can lead to changes in the expression patterns of hundreds or even thousands of genes. While it is likely that a few specific targets play pivotal roles in disease phenotypes, the possibility exists that the combined effects of multiple targets within the same pathway contribute synergistically to the overall outcome. Therefore, in addition to strategies aimed at correcting the mis-splicing of specific genes, it is equally important to explore approaches that restore the broader functional integrity of RBPs or spliceosomes, or that rescue cellular pathways enriched with defective genes.

### Alternative polyadenylation

Another mechanism that promotes transcript diversity is alternative polyadenylation (APA). Alternative isoforms with different lengths of 3’-UTRs can be produced via the use of APA sites (Tian and Manley, 2017). APA sites can also be found occasionally in intronic regions. Over 70% of mRNA-encoding genes exhibit APA isoforms (Tian and Manley, 2017). APA can regulate gene expression via different RNA processing pathways, including regulation of mRNA stability, nuclear export, translation, and subcellular localization. Aberrant use of APA sites can result in truncated mRNAs and abnormal protein expression (Passmore and Coller, 2022; Tian and Manley, 2017).

Widespread APA changes have been reported from large cohort studies of both C9ORF72-ALS and sporadic ALS brains (McKeever et al, 2023; Prudencio et al, 2015; Zeng et al, 2024), indicating a global trend toward distal 3’-UTR APA usage in ALS (McKeever et al, 2023) (Fig. 1). Though less abundant, intronic APAs also show an increase in ALS (McKeever et al, 2023), which could potentially lead to truncated mRNAs, interfering with functional protein levels. Several ALS-related genes have isoforms with different lengths of 3’-UTRs due to APA, such as *TDP-43, MATR3, SETX*, *ANXA11*, and *TIA1* (McKeever et al, 2023). Through pathway enrichment, it is speculated that lengthened transcripts might influence organellar assembly and protein localization, whereas shortened transcripts might influence transcription and protein complex assembly (McKeever et al, 2023).

APA is known to be regulated by RBPs (Passmore and Coller, 2022; Tian and Manley, 2017). For example, knockdown of TDP-43 or disease-causative mutations of TDP-43 affect APA, which tends to favor the usage of distal sites (Arnold et al, 2024; Bryce-Smith et al, 2024; Polymenidou et al, 2011; Rot et al, 2017; Zeng et al, 2024). The extended 3’-UTR is considered to increase the stability of the transcripts, such as microtubule affinity-regulating kinase 3 (MARK3), the stabilization of which is suggested to increase the accumulation of phosphorylated tau (Arnold et al, 2024). APA and stabilization of multiple transcription factor-encoding transcripts could potentially have a broader influence on transcription (Bryce-Smith et al, 2024). Several disease-relevant genes, such as *ELP1*, *NEFL*, *TMEM106B*, were also reported to have longer 3’-UTRs upon TDP-43 reduction (Zeng et al, 2024). In addition, a recent study used single-nucleus RNA-sequencing in both familial and sporadic ALS, identified cell type-specific APA dysregulation in ALS, and applied a deep learning method to identify potential *cis*/*trans* regulators of APA in disease (McKeever et al, 2023). The approach was validated by the identification of known regulators, such as TDP-43, and further revealed several splicing factors, including some with known APA functions in cancer, such as HNRNPC, SFPQ, and SRSF7. HNRNPC knockdown is known to promote distal polyadenylation site usage in cancer (Fischl et al, 2019), and its expression is downregulated in excitatory neurons of C9ORF72-ALS (McKeever et al, 2023). However, further investigation will be needed to confirm and further characterize the roles of those predicted RBPs on APAs in ALS.

Beyond ALS, APA is considered to potentially impact the function of risk genes in several neurodegenerative diseases. A recent study reported on a 3’-UTR APA transcriptome-wide genomic study in 11 brain disorders, and nominated a list of disease-associated APA-related genes, including *SNCA* in PD. It was found that 3’-extended usage of *SNCA* increases PD risk (Cui et al, 2023). RNA-seq analysis of data from AD, PD, and ALS suggests that genes with disease-specific dysregulation of APA are enriched in pathways related to protein turnover and mitochondrial function (Patel et al, 2019).

These studies affirmed an association of APA with pathological proteins and variants of neurodegenerative diseases. To enhance the understanding of APA, there is a need for improved bioinformatic tools, larger sample sizes, and functional studies investigating the relationships between APA, RBPs and disease variants. Moreover, the biological functions and pathological consequences of APA aberrations remain largely speculative, and thus need further experimental elucidation.

### RNA degradation

RNA degradation controls the steady-state pool of RNA levels at precise stage and location, and ensures the fidelity of RNA transcripts. When errors are introduced during transcription or RNA processing, different RNA-degradation pathways will be triggered to degrade the aberrant transcripts and avoid the production of defective proteins. For example, transcripts containing premature translation termination codons (PTCs) can be degraded by the nonsense-mediated decay (NMD) machinery (Daar and Maquat, 1988; Maquat, 2004). For transcripts lacking stop codons, which are usually caused by transcriptional errors, a Ski complex-mediated non-stop decay machinery will be involved (Frischmeyer et al, 2002; Garneau et al, 2007; van Hoof et al, 2002). When translation stalls on RNA transcripts, a no-go decay machinery will be engaged where binding of Pelota and HBS1L near the stalling sites will recruit the exosome and Xrn1 for endonucleolytic cleavage and decay (Doma and Parker, 2006; Garneau et al, 2007; Ikeuchi et al, 2016).

Abnormal global RNA stability has been reported in several neurodegenerative diseases (Figs. 1 and 3). A trend of mRNA destabilization is recognized in ALS fibroblasts and induced pluripotent stem cells (iPSCs) of C9ORF72-ALS (Tank et al, 2018). Accumulation of TDP-43 was proposed to account for the mRNA destabilization in the C9ORF72-ALS iPSC model, as a considerable proportion of destabilized transcripts are shared between iPSCs with overexpression of TDP-43 and iPSCs of C9ORF72-ALS (Tank et al, 2018). However, whether the iPSC model of C9ORF72-ALS exhibits TDP-43 overexpression, and whether the mechanism is conserved in neurons is unclear. A recent publication reported globally stabilized mRNAs in C9ORF72-ALS/FTD patient iPSC-derived neurons (iPSNs) and postmortem brain tissues (Li et al, 2023). It was found that the m^6^A RNA modification reduction in patient iPSNs mediates the decreased decay of m^6^A-marked transcripts, which are enriched in neuronal functions and synaptic activity (Li et al, 2023). Elevated accumulation of neuronal transcripts may potentially contribute to the hyperexcitability of ALS neurons.

Dysfunction of the NMD pathway has been implicated in several neurodegenerative diseases (Fig. 3). Overexpression of UPF1, an RNA helicase critical for NMD (Leeds et al, 1991), has been shown to have beneficial effects on reducing the toxicities induced by FUS, TDP-43, and *C9ORF72* repeat expansion, though the exact mechanisms vary (Barmada et al, 2015; Jackson et al, 2015; Ju et al, 2011; Ortega et al, 2020; Sun et al, 2020; Xu et al, 2019; Zaepfel et al, 2021). In cells with FUS and TDP-43 mutation and aggregation, UPF1 overexpression enhances the degradation of mis-spliced transcripts, including cryptic exons induced by TDP-43 loss of function, thereby reducing the potential neurotoxicity (Barmada et al, 2015; Kamelgarn et al, 2018). Overexpression studies suggest that UPF1 was insufficient in the diseased neurons; however, direct assessment of UPF1 expression has yielded controversial results. In C9ORF72-ALS/FTD, hyperactivation, inhibition or no changes in the NMD pathway have all been reported, and the protective role of UPF1 has been proposed to be exerted through NMD-mediated or NMD-independent mechanisms (Ortega et al, 2020; Sun et al, 2020; Zaepfel et al, 2021). Future studies comparing different models/cell types, and using an increased number of patient samples will help clarify these discrepancies. In addition, a recent study shows that UPF1 and UPF2 are both required for the degradation of some of the TDP-43-dependent PTC-containing mRNAs, suggesting a more complicated NMD mechanism with potentially compensatory components in the pathway (Alessandrini et al, 2024).

In addition, the membraneless RNP granules are also important for RNA degradation. Processing bodies (P-bodies) are cytoplasmic RNP granules primarily composed of components of the mRNA decay machinery and translationally repressed mRNAs (Fig. 3). It is generally believed that mRNAs recruited to P-bodies are destined for translational repression and degradation (Blake et al, 2024; Decker and Parker, 2012). The assembly and function of P-bodies have been shown to be perturbed in some neurodegenerative diseases. PD-causative mutation and cytoplasmic aggregation of alpha-synuclein directly modulate P-bodies by binding to multiple proteins in the P-body decapping module (e.g., DCP1/2, XRN1, and EDC3) on the EDC4 scaffold (Hallacli et al, 2022). Pathological alpha-synuclein in human neurons perturbs decapping module composition in P-bodies and disrupts mRNA decay kinetics, resulting in global stabilization of mRNAs (Hallacli et al, 2022). FUS does not localize to P-bodies, but it has been found that overexpression of ALS-causative mutant FUS can reduce the number of P-bodies (Takanashi and Yamaguchi, 2014). However, the RNA decay rate was not directly measured in that study (Takanashi and Yamaguchi, 2014); therefore, it remains unknown whether changes in P-body number due to FUS mutation functionally influence RNA decay.

In general, RNA degradation dysregulation in neurogenerative diseases is less extensively studied compared to other RNA processing pathways. The molecular mechanisms underlying these abnormalities often remain unclear, and many mRNA decay pathways have been rarely investigated beyond NMD. It is also noted that alterations in RNA stability under disease conditions may differ among cell types and/or vary across specific substrates. Future studies are needed to unravel the dysregulation mechanisms of different RNA decay pathways and further elucidate their roles in neuronal dysfunction and degeneration.

### RNA translation

RNA translation completes the life cycle of an mRNA, yielding the protein for the ultimate function. The efficiency of RNA translation can be influenced by intrinsic RNA structure, RBPs, translation factors, and cell signaling pathways. Translation of RNA in the context of neurodegenerative diseases with a focus on ALS has been recently reviewed elsewhere (Wang and Sun, 2023).

Global translation inhibition is generally found in AD, PD, ALS, FTD, and HD (Wang and Sun, 2023) (Fig. 1). Interaction of disease-linked proteins with core components of the mRNA translation machinery is a common mechanism. In C9ORF72-ALS/FTD, arginine-containing dipeptide repeat proteins (R-DPRs), poly-GR and poly-PR, bind to ribosomal subunits, inhibiting global protein synthesis (Hartmann et al, 2018; Kanekura et al, 2016; Loveland et al, 2022; Moens et al, 2019; Zhang et al, 2018b). It was shown by cryogenic electron microscopy (cryo-EM) that poly-GR and poly-PR block the polypeptide tunnel and the peptidyl transferase center, thereby inhibiting protein synthesis (Loveland et al, 2022). R-DPRs have also been shown to bind ribosomal RNA with a predicted affinity stronger than their binding to ribosomal proteins (Ortega et al, 2023). Furthermore, it was recently discovered that poly-GR perturbs translation elongation, increases ribosome collisions, and induces ribotoxic stress response, contributing to neurodegeneration of C9ORF72-ALS/FTD neurons (Dong et al, 2024). In HD, the aggregation-prone polyQ-expanded Htt protein shows stronger interaction with ribosomal proteins than its soluble wild-type counterpart, inducing dose-dependent inhibition of global translation that is likely to result from a combination of defective ribosome biogenesis and ribosomal stalling/collision during elongation (Aviner et al, 2024; Culver et al, 2012; Eshraghi et al, 2021; Kim et al, 2016). Another neurodegeneration-associated protein, SMN, has been reported to prime ribosomes to a considerable subset of mRNAs, regulating their translation initiation. In SMA with loss of SMN function, ribosomes are depleted from the specific subset of mRNAs, resulting in inhibited translation (Bernabo et al, 2017; Lauria et al, 2020).

Global translation inhibition due to the activation of the integrated stress response (ISR) is widely found in neurodegenerative diseases (Storkebaum et al, 2023; Wang and Sun, 2023). The ISR signaling pathway allows cells to alter the protein expression to cope with cellular stress. It can be activated by several neurodegeneration-associated types of cellular stress such as oxidative stress, endoplasmic reticulum stress, proteotoxic stress, and neuroinflammation (Wang and Sun, 2023). Phosphorylation of eukaryotic translation initiation factor eIF2α is a signature of ISR activation. This phosphorylation can be mediated by five eIF2α kinases: PERK, GCN2, PKR, HRI, and the recently identified MARK2 (Chen et al, 1991; Dever et al, 1992; Harding et al, 1999; Lu et al, 2021; Prostko et al, 1995; Storkebaum et al, 2023; Wang and Sun, 2023). Phosphorylation of eIF2α is elevated in AD patients, and suppression of the upstream kinases alleviates plasticity and cognition deficits in AD mouse models (Devi and Ohno, 2014; Lourenco et al, 2013; Ma et al, 2013; Segev et al, 2015; Tible et al, 2019). In C9ORF72-ALS/FTD, phosphorylation of eIF2α is also involved in the repeat-associated non-AUG translation of the microsatellite repeat expansion (Cheng et al, 2018; Green et al, 2017; Westergard et al, 2019). The kinase PKR and PERK have been suggested to be activated by the repeat RNA or poly-dipeptides (Zhang et al, 2014; Zu et al, 2020). In superoxide dismutase 1 (SOD1)-associated ALS, the mutant SOD1 protein is misfolded and forms aggregates, which trigger the unfolded protein response and endoplasmic reticulum stress, leading to PERK and PKR-mediated ISRs (Lindberg et al, 2005; Nishitoh et al, 2008; Saxena et al, 2009; Sun et al, 2015b). MARK2 is a newly identified kinase that mediates eIF2α phosphorylation independently of all the other kinases in response to proteotoxic stress, such as protein misfolding. It was found that G85R and A4V mutations of SOD1 can activate MARK2-mediated ISRs, in addition to the PERK-mediated ISRs (Lu et al, 2021). In FUS-ALS, phosphorylation of eIF2α is elevated in axons, suggesting local activation of ISR with early FUS pathologies (Lopez-Erauskin et al, 2018).

Global translation inhibition is a well-characterized feature of several neurodegenerative diseases. The fact that the same disease-causative RBPs and mutations lead to both global translation inhibition and RNA decay inefficiency causes a highly imbalanced stoichiometry between proteins and mRNAs. In addition, immediate ISR protects neuronal cells in response to stress, while persistent activation of ISR is harmful in neurodegenerative diseases. Future studies should investigate whether cell type specificity exists in the ISR persistence, and if inhibiting ISR could abrogate neurotoxicity in neurodegenerative diseases.

### Noncoding RNAs

In addition to protein-coding mRNAs, noncoding RNAs have also emerged as a relevant and evolving area of study in neurodegenerative diseases (Fig. 1). Noncoding RNA (ncRNA) constitutes a large and diverse domain, representing one of the major discoveries in the post-genomic era. The draft annotation of the human genome assembly T2T-CHM13 suggested the presence of over 43,000 genes of ncRNAs (>147,000 transcripts), which is about twice as many as the protein-coding genes/transcripts (Nurk et al, 2022). Loss-of-function studies have provided profound insights into the roles of ncRNAs in brain and neuronal function (Fatica and Bozzoni, 2014; Liu et al, 2017b; Sauvageau et al, 2013; Yang et al, 2021). Those ncRNAs include long noncoding RNAs (lncRNAs), microRNAs (miRNAs), circular RNAs (circRNAs), ribosomal RNAs, transfer RNAs, and many more. Here, we will focus on lncRNAs, miRNAs, and circRNAs because they have predominant regulatory functions and have been suggested to have potential functions in neurodegenerative diseases.

#### Long noncoding RNAs (lncRNAs)

One feature of lncRNAs is that their expression, especially that of the long intergenic noncoding RNAs (lincRNAs), is strikingly tissue-specific, even more so than that of protein-coding mRNAs (Cabili et al, 2011). Currently, most of the lncRNA studies in the nervous system have focused on diseases related to neurodevelopment. Our understanding of the lncRNA mechanisms in neurodegenerative diseases is relatively more limited, and whether the function of lncRNAs in disease is deleterious or protective is controversial. Some well-documented examples of lncRNAs in the context of neurodegenerative diseases are nuclear paraspeckle assembly transcript 1 (NEAT1), Homeobox transcript antisense RNA (HOTAIR), and MALAT1.

NEAT1 is one of the most abundant lncRNAs in the nucleus, essential in paraspeckle formation (An et al, 2018). In PD, elevated NEAT1 levels have been reported in postmortem brains (Kraus et al, 2017; Simchovitz et al, 2019) and peripheral blood cells of PD patients (Boros et al, 2020). Multiple functions of NEAT1 elevation have been proposed. NEAT1 was found to stabilize the disease-causing PTEN-induced putative kinase 1 (PINK1), and to increase the accumulation in the mitochondrial membrane compartment of PINK1 that triggers autophagy (Pickrell and Youle, 2015). NEAT1 upregulation also promoted the expression of another PD-causing gene, SNCA, resulting in apoptosis (Liu and Lu, 2018). In addition, NEAT1 upregulation led to neuroinflammation through a number of pathways engaging the sponging of multiple microRNAs (Boros et al, 2021). In ALS and FTD, NEAT1 was found to associate with observed paraspeckle alterations in ALS/FTD patients with TDP-43 and FUS pathologies (Wang et al, 2020).

HOTAIR is a trans-acting lncRNA engaging in chromatin remodeling and epigenetic regulation (Raju et al, 2023). Increased levels of HOTAIR have been found in in vitro and in vivo models of PD. HOTAIR increase led to decreased expression of miR-221-3p and upregulation of the microRNA targets NPTX2 and α-synuclein, triggering the secretion of inflammatory cytokines and degeneration of dopaminergic neurons (Lang et al, 2020; Sun et al, 2022). A study also suggested a direct link between HOTAIR and the stabilization of the mRNA of a key pathological gene of PD, *LRRK2* (Wang et al, 2017).

MALAT1 is a highly expressed lncRNA, localized at nuclear speckles (Arun et al, 2020). MALAT1 is increased in PD iPSNs (Abrishamdar et al, 2022). It was demonstrated that MALAT1 upregulation stabilizes α-synuclein and can increase SNCA level (Xia et al, 2019; Zhang et al, 2016a).

#### microRNAs

miRNAs are evolutionarily conserved short RNAs with ~22 nucleotides in length, encoded by DNA stretches in either intergenic or intragenic regions (Gebert and MacRae, 2019). miRNAs can repress target gene expression by affecting either mRNA degradation or translational repression (Fabian et al, 2010; Gebert and MacRae, 2019). Even though their functions have been more extensively characterized in development, miRNAs are also important in regulating gene expression for neuron function in adulthood (McNeill and Van Vactor, 2012).

miR-7 is highly expressed in the brain (Farh et al, 2005). miR-7 binds to the 3’-UTR of α-synuclein mRNA. It is decreased in the substantia nigra of PD patients and of both in vivo and in vitro models of PD (Junn et al, 2009; McMillan et al, 2017). Loss of miR-7 leads to increased expression of α-synuclein, correlating with a loss of nigral dopaminergic neurons (McMillan et al, 2017). Introducing miR-7 to an MPTP-induced neurotoxin cell culture model of PD, a commonly used PD model due to the selective effect of MPTP on dopaminergic neurons (Olanow and Tatton, 1999; Onofrj and Ghilardi, 1990), leads to downregulation of α-synuclein expression and shows a protective effect against oxidative stress (Junn et al, 2009; Li et al, 2016).

miR-133b was identified by a miRNA profiling analysis comparing the midbrains of PD patients and normal controls. This miRNA is specifically expressed in the dopaminergic neurons of midbrains and is drastically downregulated in PD patients. Overexpression of miR-133b in dopaminergic neurons suppresses maturation and dopamine release (Kim et al, 2007). Conversely, it was elevated in the plasma of a large cohort of PD patients and controls (Chen et al, 2021). The discrepancy between these studies could be attributable to the cell-type specificity of miRNA. In addition, the observed downregulation could result from the loss of dopaminergic neurons in the patients’ brains. Therefore, further functional investigations will be needed to elucidate the role of miR-133b in PD.

miR-128 is expressed in adult neurons. Mice deficient in miR-128 develop neuroexcitability and fatal epilepsy due to miR-128 effects on the regulation of numerous genes involving ion channels, transporters, and neurotransmission (Tan et al, 2013). Overexpression of miR-128 leads to reduced motor activity and alleviation of motor abnormalities associated with PD-like disease and seizures in mice (Tan et al, 2013). The effects of miR-128 on neuroexcitability are likely through the regulation of various ion channels and the ERK2 signaling pathway (Tan et al, 2013). miR-128 downregulation has also been reported in HD patient brains and HD models (Kocerha et al, 2014; Lee et al, 2011; Marti et al, 2010). Targets of miR-128 include the key pathological HD gene *Htt*, and its regulators such as *HIP1* and SP1 (Kocerha et al, 2014).

#### Circular RNAs (circRNAs)

Circular RNA (circRNA) has been extensively studied in the last few years and is increasingly linked to various neurodegenerative diseases. CircRNAs are products of back-splicing events, wherein the downstream 5′ splice site of a precursor mRNA is ligated to the upstream 3′ splice site by a 3′-5′ phosphodiester bond, forming an RNA circle (Li et al, 2018). Alternatively, circular RNAs can also be generated from intron lariats that somehow escape debranching and degradation (Lasda and Parker, 2014). These circular intronic RNAs (ciRNAs) are generally less abundant than the exon-derived ones and relatively less well-studied. Over 11,000 circRNAs are expressed in the human brain (Dong et al, 2023). They are highly expressed in the nervous system and exhibit age-dependent accumulation, which could be partially due to their structural resistance to exonucleases (Gruner et al, 2016; Kim et al, 2021; Westholm et al, 2014). CircRNAs can cause changes in chromatin, transcription, splicing, and expression of their cognate linear mRNAs, as well as serve as sponges for microRNAs (Li et al, 2018; Meng et al, 2017).

Metadata generated from the Knight Alzheimer Disease Research Center (Knight ADRC) allowed the identification of 164 cortical circRNAs with significant association with AD traits, including AD diagnosis, pathologies, and co-expression with AD genes (Dube et al, 2019). The changes in circRNA levels differ from the expression changes of their cognate linear mRNAs. Among them, several circRNAs contain binding sites for microRNAs. For example, circHOMER1, the levels of which decrease with increasing dementia severity, possesses five predicted binding sites for miR-651, whose downstream targets include the AD-related genes PSEN1 and PSEN2, suggesting a potential sponging mechanism of this circRNA (Dube et al, 2019). The BRAINcode project revealed that 29% of Parkinson’s and 12% of Alzheimer’s disease-associated genes produce circRNAs (Dong et al, 2023). Studies of specific circRNAs, such as circPSEN1, showed differential expression in autosomal-dominant individuals caused by pathogenic mutations in *APP, PSEN1*, and *PSEN2* (Chen et al, 2022a).

In ALS, FUS regulates the biogenesis of circRNAs by binding near the back-splicing junctions (Errichelli et al, 2017). In HD, the CAG microsatellite repeat expansion has been found to suppress circRNA biogenesis in mouse neural progenitor cells (Ayyildiz et al, 2023). Future studies should investigate whether circRNA levels are reduced in HD patients. Moreover, a study suggested the potential of using circRNA species as biomarkers for ALS in blood samples (Dolinar et al, 2019), which needs further validation.

The factors contributing to the changes in circRNAs in these neurodegenerative diseases have not been fully elucidated. One hypothesis is that the age-associated circRNA expression arises from a compromised splicing machinery, which suggests an interplay between different disease-related molecular pathways. This is supported by evidence indicating that depletion of spliceosomal components or treatment with splicing inhibitors can increase circRNA biogenesis from back-splicing events (Liang et al, 2017a). Alternatively, a recent study reported that increased transcriptional elongation speed (RNA polymerase II speed) is associated with elevated formation of circRNAs during aging (Debes et al, 2023), which is consistent with a previous study on the correlation between parental gene transcription elongation and circRNA back-splicing (Zhang et al, 2016b).

Despite substantial progress in characterizing the potential functions of ncRNAs in neurodegenerative diseases, our understanding is still limited. Building on the observed correlations, it is important to understand how dysregulated ncRNAs contribute to the phenotypes of the diseases. Further functional studies will serve as crucial missing pieces of evidence. As noted in some examples above, ncRNAs have high cell specificity. Therefore, cell types should be taken into consideration when pursuing functional studies. Implementation of single-cell sequencing or spatial transcriptomic approaches could help understand the cell type distinction and spatial molecular interactions of ncRNAs in normal and disease contexts.

### RNA modifications

Over 160 chemical modifications have been identified in RNA molecules to date (Boccaletto et al, 2022). Any RNA molecule possesses at least one modification at some point during its life cycle. RNA modifications can impact almost all stages of the RNA life cycle. Chemical modifications on single nucleotides can alter the electrostatic charges of the RNA molecules, potentially influencing their phase separation properties. Ribosomal RNA (rRNA) and transfer RNA (tRNA) are thought to be the most heavily modified RNAs. Such modifications are usually not reversible, and they are critical for the RNA structures (Sloan et al, 2017). There has been an increasing number of different types of modification found in mRNAs as well as in regulatory noncoding RNAs. The most common ones on purine or pyrimidine bases are methylation, pseudouridylation, and adenosine-to-inosine (A-to-I) editing (Delaunay et al, 2023). These modifications are reversible and play important roles in various steps of RNA processing regulation (Roundtree et al, 2017). Lately, pathological aberrant RNA modifications have emerged as critically involved in neurodegenerative diseases (Fig. 1).

*N*^*6*^-methyladenosine (m^6^A) is the most prevalent internal mRNA modification in eukaryotic mRNAs (Dominissini et al, 2012). It is one of the few reversible RNA modifications that regulates RNA metabolism and abundance in the nervous system (Fan et al, 2023; Roundtree et al, 2017). m^6^A is installed by the “writer” methyltransferase complex composed of the core subunits METTL3 and METTL14, and it can be removed by the “eraser” protein FTO or ALKBH5 demethylase (Dominissini et al, 2012). “Reader” proteins selectively recognize m^6^A-marked RNA and determine the fate of these RNAs. A number of studies have demonstrated the important roles of m^6^A in regulating brain function, from development and synaptic plasticity to learning, memory, and neurodegeneration (Livneh et al, 2020; Wang et al, 2018). We recently found that m^6^A is globally reduced in poly-A RNAs in C9ORF72-ALS/FTD iPSC-neurons and postmortem brains, resulting in transcriptome-wide mRNA stabilization and gene expression elevation in the patient iPSC-derived neurons and postmortem motor cortex, particularly for genes involved in synaptic activity and neuronal function (Li et al, 2023). Moreover, m^6^A modification upstream of *C9ORF72* repeat expansion regulates the decay of both sense and antisense repeat RNA. Rescuing m^6^A modification in the patient neurons alleviated pathologies and improved neuron survival (Li et al, 2023). TDP-43 was reported to bind to m^6^A-marked RNAs (McMillan et al, 2023). Knockout of m^6^A reader YTHDF2 mitigates neurotoxicity in TDP-43-overexpressing primary neurons (McMillan et al, 2023). In sporadic ALS, both downregulation and upregulation of m^6^A modification levels have been reported in different reports. These studies used immunohistochemistry approaches to evaluate the global m^6^A signal, which cannot distinguish the contributions from structural RNAs (such as rRNAs) and mRNAs (Martin et al, 2022; McMillan et al, 2023). The discrepancy in the results suggests that either this staining approach is not quantitative, or that there is high heterogeneity among sporadic cases. It is possible that there are sub-groups of sporadic ALS cases that show different m^6^A changes induced by distinct molecular mechanisms. This requires further studies with larger numbers of controls and disease samples to be clarified.

The m^6^A methyltransferase METTL3 was reported to be reduced in AD brains (Castro-Hernandez et al, 2023; Huang et al, 2020; Zhao et al, 2021), which mirrored observations from primary cortical neurons treated with soluble Aβ oligomers (Zhao et al, 2021). Moreover, METTL3 knockdown in the mouse hippocampus induced oxidative stress, DNA damage, spine loss and neurodegeneration, and mouse cognitive behavior deficits (Zhao et al, 2021). Another study found that oligomeric tau induced the cytoplasmic translocation of the m^6^A reader protein hnRNPA2B1 and the cytoplasmic mislocalization of m^6^A (Jiang et al, 2021). Global downregulation of m^6^A was also reported in a PD-related cell model and rat brains. Reduction of m^6^A increased the expression of N-methyl-d-aspartate (NMDA) receptor 1, elevated calcium influx, and contributed to dopaminergic neurodegeneration (Chen et al, 2019). Moreover, a recent study found that abnormally expressed transcripts in the HD mouse brain had reduced m^6^A levels, particularly adjacent to TDP-43-binding sites, which likely contributes to the alternative splicing changes in HD (Nguyen et al, 2023).

*N*^*1*^-methyladenosine (m^1^A) is highly abundant in structural RNAs, and present at low levels in mRNAs, where it is less abundant than m^6^A. m^1^A is usually near the translation starting site (Dominissini et al, 2016). A recent study identified m^1^A modification in the CAG repeat RNA, and its binding to TDP-43 contributes to the cytoplasmic mislocalization and aggregation of TDP-43 (Sun et al, 2023).

Pseudouridine (Ψ), an isomer of uridine, is present in noncoding RNAs (i.e., tRNA, rRNA, and snRNA) and in mRNAs (Schwartz et al, 2014). Unlike m^6^A, which is enriched at the stop codon of mRNAs, pseudouridines are distributed along the whole length of the mRNAs from the 5′- to the 3′-UTR without any region-specific enrichment (Khoddami et al, 2019; Schwartz et al, 2014). The functions of pseudouridine include stabilizing RNA structures and destabilizing interactions with RBPs (reviewed in (Borchardt et al, 2020)). In myotonic dystrophy type 2 (DM2), a neuromuscular disease involving neuronal loss and global neuronal impairment, pseudouridines within the CCUG repeats in the intron of *CNBP* decrease repeat RNA dynamics and thus reduce sequestration of MBNL1 to the repeat RNA (deLorimier et al, 2017). The link between pseudouridines and neurodegenerative diseases has not been extensively studied. It was demonstrated that acute oxidative stress in cells could trigger a significant elevation of pseudouridine levels in mRNAs (Feiler et al, 2015). It would be interesting to examine pseudouridine changes during aging or under specific pathological conditions.

A-to-I editing converts adenosines to inosines in both coding and noncoding RNAs. It is catalyzed by adenosine deaminases acting on RNA (ADARs), which are found to be enriched in the nervous system. A-to-I editing can affect base pairing, alter codons, and change splice sites. A recent computational trait-association study suggested that ADAR-mediated A-to-I editing in double-stranded regulatory RNA may underlie multiple neurodegenerative diseases, including AD, PD, and ALS (Li et al, 2022). An editing defect of the *GluR2* mRNA has been found in ALS spinal motor neurons due to the downregulation of ADAR2 (Hideyama et al, 2012; Kawahara et al, 2004; Kawahara et al, 2003; Takuma et al, 1999). The reduced editing at the Q/R site of GluR2 leads to increased calcium influx and excitotoxicity to motor neurons, eventually triggering neuronal death (Kawahara et al, 2004; Kawahara et al, 2003; Takuma et al, 1999). Moreover, the increased calcium influx activates the calcium-dependent cysteine protease calpain, which cleaves TDP-43 and produces C-terminal fragments that mis-localize and aggregate in the cytoplasm (Yamashita et al, 2012). In addition, ADAR2 is reported to be mislocalized in C9ORF72-ALS/FTD, leading to widespread RNA editing aberrations, especially in the pathways of integrated stress response and EIF2 signaling (Moore et al, 2019). The Q/R editing of *GluR2* mRNA was also found to be reduced in the hippocampus of AD patients (Akbarian et al, 1995; Gaisler-Salomon et al, 2014; Khermesh et al, 2016), which was recently suggested to potentially function as an epigenetic switch that regulates dendritic spines and links to neurodegeneration and memory deficits in AD (Wright et al, 2023). In synucleinopathy-associated neurodegenerative diseases, such as PD and dementia with Lewy bodies (DLB), the expression of the editing inhibitor ADAR3 is reduced by transcription inhibition due to the insolubility of NONO/SFPQ, thereby increasing the editing on many transcripts encoding axonal, synaptic and mitochondrial proteins. These aberrantly edited RNAs are retained in the nucleus, leading to reduced protein levels (Belur et al, 2024).

The understanding of RNA modifications in neurodegenerative diseases has been expanding rapidly in the past few years. Studies of RNA modifications, also referred to as epitranscriptomics, provide a valuable angle to study neurodegenerative diseases beyond the primary genetic code. As in the above examples, RNA modification changes can influence multiple steps of RNA metabolism, including RNA decay and RBP localization. In addition, due to the prevalence of some RNA modifications, the impact of such modifications is likely to be broad on the transcriptome. It coincides with the technological developments that enable the measurement of RNA modifications with increased resolution and accuracy. The diversity of different types of RNA modifications is increasingly recognized in neurodegenerative diseases. In addition to deciphering the relationship between individual RNA modifications and disease physiology and pathology, it will be equally important to understand the interactions among different RNA modifications in the future. Moreover, the key mechanisms driving age-related or disease-specific epitranscriptomic changes remain largely unexplored. Performing more basic research to address the role of RNA modifications during aging, in different brain regions and cell types, will be helpful to enhance our understanding of neurodegenerative diseases.

### Nucleocytoplasmic transport of RBPs

Many RBPs are predominantly localized in the nucleus, but also constantly shuttle between the nucleus and the cytoplasm, participating in multiple steps of RNA processing. However, it has been found in many neurodegenerative diseases that RBP proteins are mislocalized to the cytoplasm, which results in the loss of their nuclear functions (Fig. 4). As many of those RBPs are aggregation-prone, they can form cytosolic inclusions and exhibit gain of toxicity. Exploring the factors contributing to the mislocalization of RBPs is crucial for understanding disease mechanisms.

The nucleocytoplasmic transport of RBPs is bidirectional. Efficient nucleocytoplasmic transport requires functional nuclear pore complexes (NPC), a Ran gradient, and nuclear transport receptors. NPCs are located on the nuclear envelope. They are composed of over 30 nucleoporins (Nups), which are arranged in an eightfold rotational symmetry (reviewed in (Khan et al, 2020)). A nucleoplasmic-cytoplasmic gradient of RanGTP–RanGDP across the nuclear envelope, generating asymmetry between the nucleoplasm and the cytoplasm, provides essential directionality to nucleocytoplasmic transport (Nachury and Weis, 1999). Nuclear transport receptors include importins and exportins (Ding and Sepehrimanesh, 2021). Importins recognize nuclear localization signals (NLS) in RBPs to transport the RBPs from the cytoplasm to the nucleus. Some well-characterized importins include importin β1, transportin 1 and 3 (TNPO1/3). Exportins (XPOs) recognize nuclear export signals (NES) in RBPs to transport the RBPs from the nucleus to the cytoplasm (Ding and Sepehrimanesh, 2021). There is also a third class of transport receptors, named biportins, which are relatively ambiguously defined. Biportins can function bidirectionally as importins or as exportins (Yang et al, 2023). Some mutations in RBPs are located in their NLS, therefore affecting the nuclear import of the protein, such as in FUS NLS mutations (Kwiatkowski et al, 2009; Mackenzie et al, 2010). Wild-type RBPs can also be mislocalized in sporadic diseases, due to NPC/transport machinery defects.

Nuclear pore complex (NPC) components have been shown to be perturbed in ALS and FTD, as evidenced by morphological irregularities and altered expression of nucleoporins. This perturbation could contribute to nucleocytoplasmic mislocalization of RBPs, including TDP-43. Different mechanisms could contribute to such nuclear pore defects. First, NPCs are susceptible to aging (Sakuma and D’Angelo, 2017). In post-mitotic cells, NPCs—especially the NPC scaffold components—are extremely long-lived with a very slow turnover rate (D’Angelo et al, 2009; Savas et al, 2012). Given the long life of NPCs, age-related damage accumulation of NPCs eventually results in increases in nuclear permeability and abnormal distribution of nuclear and cytoplasmic proteins in aged neurons (D’Angelo et al, 2009). Second, specific mutations or pathologies can cause impairment of the nuclear pore complex. For example, the expression of *C9ORF72* microsatellite repeat expansion RNA and proteins is associated with NPC defects (Coyne et al, 2021; Freibaum et al, 2015), partially due to increased nuclear expression and localization of CHMP7, an NPC quality control protein that impacts NPC homeostasis on the nuclear envelope and leads to TDP-43 leakage into the cytoplasm (Coyne et al, 2020).

In AD, pathological phospho-tau can directly interact with NPC components and trigger mislocalization of some Nups, thus disrupting the NPC function and compromising the NPC diffusion barrier (Eftekharzadeh et al, 2018; Lester and Parker, 2018). In HD, the expression of microsatellite CAG repeat expansions in the *huntingtin* (*htt*) gene results in morphological changes of NPCs and consequently mRNA nuclear retention (Gasset-Rosa et al, 2017). In addition, the aggregates formed by polyglutamine-rich proteins are found to co-aggregate with NPC components or trigger the mislocalization of NPC components to stress granules, which influences the solubility and functionality of NPCs (Gasset-Rosa et al, 2017; Grima et al, 2017; Shi et al, 2017; Zhang et al, 2018a). Moreover, the aberrant phase transition of RBPs, which is discussed in more detail in the next section, can also influence the NPC function. For example, it was found that the insoluble TDP-43 cytoplasmic inclusions could potentially sequester nucleoporins and transport factors, and even trigger the mislocalization of some of the nucleoporin proteins (Chou et al, 2018; Khalil et al, 2022).

Disrupted Ran gradient and nuclear transport receptors have also been reported in some neurodegenerative diseases. Huntingtin-linked polyglutamine induces cytoplasmic mislocalization of RanGAP1, a regulatory protein that hydrolyses Ran-GTP to Ran-GDP (Gasset-Rosa et al, 2017). Poly-GA is reported to impair the importin-α/β-dependent pathway and lead to compromised import of TDP-43 (Khosravi et al, 2017). In AD hippocampal neurons, both RanGDP transporter and importins have been reported to be mislocalized to the cytoplasm (Lee et al, 2006; Sheffield et al, 2006). The link between Ran gradient dysregulation and neurodegeneration is best-characterized in C9ORF72-ALS/FTD. The Ran gradient and its associated regulatory proteins such as RCC1 are disrupted in multiple C9ORF72-associated ALS/FTD models, including long differentiated iPSC-neurons, induced neurons of carriers, and *Drosophila* models expressing G_4_C_2_ repeats (Freibaum et al, 2015; Jovicic et al, 2015; Klebe et al, 1995; Zetsche et al, 2015). Nuclear mislocalization of RanGAP1 has been observed in C9ORF72-ALS/FTD postmortem motor cortex, and overexpression of RanGAP1 rescued the impaired Ran gradient in patient-derived neurons (Zetsche et al, 2015). Antisense oligonucleotides (ASOs) targeting *C9ORF72* sense or antisense repeat RNAs have been shown to attenuate nucleocytoplasmic transport abnormalities and NPC defects linked to some of the Nups (Coyne et al, 2020; Rothstein et al, 2023; Zetsche et al, 2015).

In addition, other mechanisms may also contribute to RBP nucleocytoplasmic mislocalization. Given the RNA-binding nature of RBPs, the localization of RBPs can be influenced by the distribution of target RNAs. Acute depletion of RNA abundance in the nucleus by transcriptional inhibition of RNA polymerase II can induce FUS cytoplasmic mislocalization (Tsai et al, 2022), as well as TDP-43 mislocalization (Duan et al, 2022). The nuclear RNA binding of TDP-43 has been shown to be important for its nuclear retention (Duan et al, 2022). As mRNAs exhibit extensive nucleocytoplasmic distribution alterations in ALS and FTD (Fernandopulle et al, 2021; Kim et al, 2017; Markmiller et al, 2021; Tsai et al, 2022), this could potentially promote RBP mislocalization.

Deficiency of nucleocytoplasmic transport provides a plausible mechanism for RBP mislocalization and RNA metabolism dysregulation. However, many important questions remain unanswered. For example, current evidence of NPC defects appears to be circumstantial. Therefore, it could be of great importance to identify the exact causes of NPC defects in neurodegenerative diseases. Beyond current evidence of expression changes in NPC components, the topology and structure of NPCs should also be investigated to better understand their dysregulated functions in disease. Furthermore, comparing NPC structure and function across different cell types will help delineate the potential mechanisms underlying neuron-specific deficits. Nucleocytoplasmic transport is a coordinated process involving events between the nucleus and the cytoplasm, and identifying which interactions are essential or which events occur early in disease progression could provide useful insights for the development of potential therapeutic strategies.

### Phase separation of RBPs

Phase separation is a local concentration change of molecules that allows the formation of membraneless condensates (Lyon et al, 2021; McDonald et al, 2020; Molliex et al, 2015). Biomolecular condensate formation is a universal phenomenon in cells, playing essential roles in various cellular processes, such as transcription, translation, and signaling (Lyon et al, 2021; McDonald et al, 2020; Molliex et al, 2015). Various membraneless RBP granules are condensates formed with both proteins and RNAs. Many RBPs contain intrinsically disordered regions (IDRs) that facilitate phase separation.

Stress granules are an extensively studied type of phase-separated granules that show links to neurodegeneration. They are formed in response to cellular stress and are composed of non-translating mRNPs (Marcelo et al, 2021; Weskamp and Barmada, 2018). They are enriched with mRNAs, RBPs, and translation initiation factors, and depleted of deadenylation proteins. Therefore, they are largely considered to maintain mRNA stability (Marcelo et al, 2021; Weskamp and Barmada, 2018). However, the exact function of stress granules has not been completely elucidated. Stress granules are transient and dynamic structures. Persistent stress granule formation or delayed stress granule disassembly are considered to contribute to protein aggregation in neurodegenerative diseases (Marcelo et al, 2021; Wolozin, 2012). This hypothesis is supported by evidence indicating that many neurodegeneration-associated proteins are found in stress granules, including TDP-43, FUS, hnRNPA1, and Tau (Baron et al, 2013; Cruz et al, 2019; Gui et al, 2019; Li et al, 2013; Liu-Yesucevitz et al, 2010). However, stress granule marker proteins, such as G3BP1 and TIA1, are rarely found to co-aggregate with these pathological protein inclusions in patient postmortem tissues. One possibility is that stress granules can increase the propensity of specific RBPs, not the whole stress granules, to form aggregates. However, the mechanism and the connection of stress granules to RBP aggregation need further elucidation.

Alternatively, RBPs can form independent condensates by themselves. Posttranslational acetylation of TDP-43 has been found to drive the demixing of TDP-43 into intranuclear liquid crystal spherical shells with liquid cores, named “anisosomes” (Yu et al, 2021). Optogenetic TDP-43 nucleation induces insoluble aggregates, which do not co-localize with stress granules (Mann et al, 2019; Otte et al, 2020). FUS phase separation in cells has been found to be modulated by posttranslational methylation (Qamar et al, 2018). In vitro experiments have shown that droplets of FUS itself can convert into fibrous structures over time, and this process can be accelerated with patient-derived FUS mutations (Murthy et al, 2019; Patel et al, 2015; Tetter et al, 2024). Time-lapse imaging revealed the nucleation process of FUS forming short fibers to long fibers (Patel et al, 2015).

The aberrant phase separation of RBPs is considered to be an important mechanism of protein aggregation in neurodegenerative diseases (Nussbacher et al, 2019) (Fig. 4). Many disease-causing mutations tend to cluster in the low complexity domains (LCDs) with intrinsically disordered regions (IDRs) (Lagier-Tourenne et al, 2010), which are tightly associated with phase separation (discussed in more detail later). These mutation clusters are found in the C-terminal domains of TDP-43 (Lagier-Tourenne et al, 2010; Nussbacher et al, 2019), hnRNPs (Kim et al, 2013), and TIA1 (Mackenzie et al, 2017), as well as in the N-terminal domains of FET family proteins FUS (Kwiatkowski et al, 2009; Vance et al, 2009), EWS (Couthouis et al, 2012) and TAF15 (Neumann et al, 2011). Failure to maintain the liquid phase homeostasis can result in RBP loss of function and influence a spectrum of protein homeostasis and cellular functions. Emerging evidence confirms that the phase separation process is a complex multifactorial transformation (Carey and Guo, 2022), with multivalence serving as a consensus mediator. The multivalence can arise from the IDRs of RBP proteins, the multivalent nature of RNAs, and versatile electrostatic interactions. In neurodegenerative diseases, multiple factors may contribute to aberrant phase separation and eventually lead to protein aggregation, such as changes in posttranslational modifications and protein homeostasis pathways during aging, mutations in IDRs that influence electrostatic interactions, and dysregulated RNAs resulting from impairment of RNA metabolism (Liu et al, 2017a; Nussbacher et al, 2019). Therefore, aberrant phase separation is likely a collective consequence of defects or changes in RBPs, RNAs, and the surrounding microenvironment. The changes in RNA metabolism and the mislocalization of RBPs discussed above may ultimately contribute to the aberrant phase separation that is widely observed in many neurodegenerative diseases, and such aberrant phase separation of RBPs could be further amplified and propagate under disease conditions.

It is now understood that proteins prone to undergo phase separation are characterized by several features, such as IDR domain, multivalency, and RNA engagement. However, there is still a lack of evidence linking phase separation to aggregation formation observed in patients. One current bottleneck in advancing this field further lies in the limited tools for monitoring and capturing in vivo phase separation and aberrant transition in neurons and neurodegenerative disease models. Recent advances in the available techniques for studying phase separation in vivo deepen our understanding of neurodegeneration-associated phase separation. Recently, several studies used optogenetic tools to visualize the nucleation process of FUS and TDP-43 in cells (Otte et al, 2020; Shimobayashi et al, 2021). Recent work using cryo-EM has revealed more precise structures and protein components of the aggregates in patient postmortem tissues, such as TAF15 (Tetter et al, 2024) and TDP-43 (Arseni et al, 2023; Arseni et al, 2024) that formed amyloid filaments in different types of FTD. Such technological advances can help elucidate the protein-protein and protein-RNA interactions, providing insights into the mechanisms of phase separation transition and RBP protein aggregation in neurodegenerative diseases.

### RNA repeat expansion and neurodegeneration

Microsatellite repeat expansion causes over 50 neurological and neuromuscular diseases, including spinocerebellar ataxia, myotonic dystrophy (DM1 and DM2), HD, and ALS/FTD (Fig. 4). RNAs with repeat expansion can induce various toxicities and contribute to disease pathogenesis, especially when the expansion is located in the noncoding regions of the gene, including the 5’- and the 3’-UTRs, and introns (Paulson, 2018). In many cases, the microsatellite repeat-expanded sequence can be bidirectionally transcribed (Gudde et al, 2017; Haeusler et al, 2016; Paulson, 2018), although the mechanism remains unclear. Transcripts from both sense and antisense strands have been detected in C9ORF72-ALS/FTD, HD, SCA7, and DM1 (Castro et al, 2020). It is usually thought that the antisense transcripts may be produced at lower levels than the sense transcripts (Paulson, 2018), but based on evidence from C9ORF72-ALS/FTD, this may not always be true. However, there is limited information on the stoichiometry of sense and antisense transcripts at the single-cell level.

The RNAs with repeat expansions can form RNA foci in the nucleus, regardless of sense or antisense origin (Paulson, 2018). The repeat RNA foci can sequester RBPs and cause their loss of function. A clear line of evidence on repeat RNAs sequestering RBPs and influencing RNA splicing refers to myotonic dystrophy type 1 and 2 (DM1/DM2) where CUG- or CCUG-repeat RNA binds MBNL1–3, muscle or neuron-enriched RBPs. Loss of MBNL function leads to global splicing and APA dysregulation of genes important for muscle or neuron development and function, contributing to the muscle and eye symptoms of patients (Fardaei et al, 2002; Kanadia et al, 2003; Lin et al, 2006; Mankodi et al, 2001; Miller et al, 2000). This mechanism has been confirmed in multiple disease models. MBNL depletion mirrors the majority of splicing defects in DM1 patients, and overexpression of MBNL significantly rescues the splicing deficits in the disease (Kanadia et al, 2003; Kanadia et al, 2006). In *C9ORF72* repeat expansion-linked ALS and FTD, the sense (GGGGCC)n and antisense (CCCCGG)n repeat expansion have also been shown to interact with RBPs. However, instead of one dominant RBP being sequestered by the repeat RNA, as seen in the DM1 case, multiple RBPs have been reported to bind to the C9ORF72 expanded repeats. These include Pur-α (Xu et al, 2013), RBPs involved in RNA transcription, editing, splicing and transport (Celona et al, 2017; Cooper-Knock et al, 2014; Donnelly et al, 2013; Haeusler et al, 2014), master regulator hnRNP family protein (Conlon et al, 2016; Cooper-Knock et al, 2014; Mori et al, 2013), and increasing paraspeckle components (Bajc Cesnik et al, 2019). More examples can be found in other reviews (Conlon and Manley, 2017; Nussbacher et al, 2019; Schwartz et al, 2021). A recent study revealed an alternative mechanism suggesting that, instead of sequestering one specific protein, the (GGGGCC)n repeat RNA affects the biophysical properties and functions of the endogenous nuclear speckles, a type of nuclear RNP granules, leading to global splicing repression (Wu et al, 2024).

Apart from the repeat RNA-mediated toxicity, the repeat peptides encoded by the RNA repeats can also show toxicity in neurons. The repeat RNAs, especially those with secondary structures, undergo non-canonical AUG-independent repeat-associated (RAN) translation in all possible reading frames. Multiple poly-peptides or poly-dipeptides can be generated from each repeat sequence, and RAN translation has been found for many repeat expansion sequences, including those in the coding region (Banez-Coronel and Ranum, 2019). The cellular toxicity of different peptide repeats varies, influenced by the amino acid composition (Freibaum and Taylor, 2017). The RAN translation can begin using the 5’ cap scanning mechanism, and can also occur independently of the 5’ cap, such as in intron-localized repeats (Wang and Sun, 2023). Many translation initiation factors and RBPs have been reported to regulate the translation efficiency of different repeats, which can influence neurotoxicity via modulating the toxic protein accumulation (Wang and Sun, 2023).

Translation elongation has also been found to be affected by some repeat sequences, especially arginine-containing peptide repeats that can be produced from GGGGCC repeats and CGG repeats (Park et al, 2021; Radwan et al, 2020; Wang and Sun, 2023; Wright et al, 2022). In C9ORF72-ALS/FTD, two-color single molecule imaging techniques were used to reveal that translation elongation of GGGGCC repeats is faster in the GA frame than in GP, and the GR frame was characterized by the lowest elongation rate (Latallo et al, 2023). It has also been reported that GGGGCC repeats produce aggregation-prone chimeric DPR species containing GA and GP (McEachin et al, 2020). In fragile X-associated tremor/ataxia syndrome (CGG repeats) and SCA6 (TGGGCC repeats), chimeric DPR species due to translational frameshifts were also detected (McEachin et al, 2020; Wright et al, 2022). Several repeat proteins have been shown to affect various RNA processing pathways, such as translation and splicing, discussed in earlier sections.

Overall, the expression of genetic repeat expansions is a prominent cause of neurodegeneration, triggering cascades of multifaceted dysregulation. Both the repeat RNA and the generated peptide repeats can perturb multiple RNA processing pathways via different mechanisms (Fig. 4). It has been challenging to determine which repeat-derived product is the critical factor in disease pathogenesis, repeat RNA or peptide, sense or antisense, and which repeat peptide. It is possible that all of these factors contribute to disease, and there might be synergistic neurotoxic effects. In addition, different species may exhibit predominant toxicity in different cell types and at various disease stages. More studies are needed to fully understand these interactions.

### Biomarkers and therapeutics

Mechanistic studies have enabled the discovery of a range of promising potential biomarkers for early detection and stratification of patients. Currently established general molecular biomarkers for neurodegenerative diseases, mainly for AD and ALS, are neurofilament light chain and its phosphorylated heavy chain in serum and cerebrospinal fluid (CSF) (Loeffler et al, 2020; Rossi et al, 2018; Staffaroni et al, 2022; Verde et al, 2019). Aβ peptides, total tau level, phosphorylated tau level, and β-synuclein are biomarkers for AD (Alcolea et al, 2023).

Recently, research on RNA metabolism has been making significant contributions to biomarker development, particularly cryptic splicing targets of TDP-43. Two recent publications are promising regarding the detection of cryptic peptides in the CSF or iPSC-neurons of ALS/FTD patients (Irwin et al, 2024; Seddighi et al, 2024). Remarkably, the TDP-43-dependent cryptic epitope of *HDGFL2* was detectable in the CSF of pre-symptomatic C9ORF72-ALS/FTD carriers (Irwin et al, 2024), suggesting that CE and its cryptic peptides may facilitate earlier diagnosis of ALS. Further studies will be necessary to explore more CE events and cryptic peptides, develop sensitive and reproducible assays to detect CE RNA and/or peptides, compare or combine CE features with established neurofilament biomarkers, and investigate their longitudinal changes during disease progression. It is expected that the development of a panel of molecular biomarkers, monitoring different molecular pathways, could provide better guidance for clinical trial design and patient stratification.

The increasing understanding of RNA functions enhances the development of RNA-targeting therapeutic approaches in neurodegenerative diseases (Fig. 5). For diseases associated with dominant gain-of-function mutations, targeted RNA-silencing approaches, such as RNA interference (RNAi) and antisense oligonucleotides (ASO), are broadly used and some have been clinically validated and approved. Here, we will focus on ASO implication in neurodegenerative diseases. Thorough discussions about siRNA drugs and small-molecule compounds can be found in other reviews (Ahn et al, 2023; Angelbello et al, 2020; Perez-Arancibia et al, 2022; Setten et al, 2019).

ASOs have emerged as a promising RNA-targeting therapeutic approach. They are DNA oligos designed to target complementary RNA sequences via Watson–Crick base pairing (Levin, 2019). The nucleotides are chemically modified to increase the stability, binding affinity, and specificity of the oligos (Bennett et al, 2021; Roberts et al, 2020). There are two modes of ASO action. Firstly, the “gapmer” ASOs, which contain a central DNA-based unmodified sequence flanked by two wings of modified nucleotides, recruit RNase H1 for the degradation of target RNAs. Secondly, steric-blocking ASOs, which are modified at all nucleotides, bind to the target RNA, affect RNA structures or block interactions with RBPs, but do not induce RNase H cleavage (Fig. 5) (Nikom and Zheng, 2023; Roberts et al, 2020).

Multiple degradative ASOs have been approved for clinical usage. For example, Tofersen is an ASO targeting mutant *SOD1* RNA for degradation to stop the production of toxic SOD1 proteins (Miller et al, 2022). Inotersen is approved for the treatment of familial amyloid polyneuropathy and cardiomyopathy via targeting transthyretin-encoding transcript for degradation (Benson et al, 2018). However, several highly anticipated ASO drugs, namely one targeting *Htt* in Huntington disease (Tominersen) and two targeting the *C9ORF72* sense repeat-containing transcript in C9ORF72-ALS/FTD (IONIS-C9Rx/BIIB078 and Wave WVE-004), all failed in clinical trials (Kingwell, 2021; Kwon, 2021; van den Berg et al, 2024), despite success in preclinical studies with mouse models (Jiang et al, 2016; Kordasiewicz et al, 2012; Liu et al, 2022; Tran et al, 2022). There could be multiple reasons that need to be explored, including toxicity contribution from the antisense repeat and haploinsufficiency, among others. In addition, the clinical trial of the ASO targeting *ATXN2* (i.e., ION541/BIIB105) (Scoles et al, 2017) was discontinued due to its limited rescue effect on neurofilament and clinical measures. Promisingly, many other ASOs are being subjected to clinical trials with encouraging evidence. For example, IONISMAPTRx/BIIB080 targeting *MAPT* for AD treatment is at the phase-2 trial stage across multiple countries. ION859/BIIB094 targeting *LRRK2* for PD treatment is at the phase-1 trial stage, and the results are expected by the end of 2024. Moreover, ASOs targeting α-Synuclein showed beneficial effects in a rodent model of PD (Cole et al, 2021).

Steric-blocking ASOs can be used for diverse purposes (Fig. 5). The most widely used application of steric-blocking ASOs is splicing modulation. The first FDA-approved ASO drug for neurological diseases is Nusinersen (Spinraza), designed to treat SMA (Finkel et al, 2017; Neil and Bisaccia, 2019). The ASO is designed to bind and increase the exon 7 inclusion of *SMN2*, which can produce elevated levels of functional SMN protein to compensate for the loss of *SMN1*, the cause of SMA (Hua et al, 2010; Hua et al, 2007; Rigo et al, 2012). Thus far, there are a number of splicing-switching ASOs approved by the FDA for different neurological diseases, including four drugs for Duchenne muscular dystrophy (i.e., eteplirsen, golodirsen, casimersen and viltolarsen) (Baker, 2017; Dhillon, 2020; Heo, 2020; Pascual-Morena et al, 2020; Shirley, 2021), and a patient-customized precision drug for the rare genetic neurodegenerative disease Batten disease (Kim et al, 2019). In addition, several steric-blocking ASOs are actively under development for various neurodegenerative diseases and have shown promising initial results. For example, ASOs were designed to inhibit cryptic exon inclusion in *STMN2* induced by loss of TDP-43, and they have been shown to restore the STMN2 protein level in mice (Baughn et al, 2023). *UNC13A* is another well-characterized TDP-43-linked cryptic exon-containing transcript. Using ASOs to inhibit the inclusion of the cryptic exon can rescue the UNC13A protein level and restore normal synaptic function (Keuss et al, 2024). For AD, ASOs that are designed to inhibit *MAPT* exon 10 inclusion have successfully achieved splicing alteration from toxic 4R to 3R in human tau-expressing mice (Schoch et al, 2016), suggesting important therapeutic potential.

Steric-blocking ASOs can also be designed for translation modulation. It is known that the 5’-UTR of an mRNA contains *cis-*acting elements that can modulate translation initiation (Chu and von der Haar, 2012). ASOs targeting the regulatory elements of the 5’-UTR showed selective effects on modulating protein translation (Hedaya et al, 2023; Liang et al, 2017b). However, no translation-modulating ASOs have been tested in neurodegenerative diseases so far. Furthermore, steric-blocking ASOs have been proposed to target aberrant miRNAs and prevent them from targeting mRNAs for decay (Lima et al, 2018).

RNA-targeting therapies exponentially broaden the spectrum of druggable targets by modulating the expression of coding and even noncoding genes. Given the wide recognition of RNA dysregulation in neurodegenerative diseases and the clinical success in some cases, RNA-targeting therapies hold great promise as candidates for precision medicine. However, several challenges remain in the clinical translation of RNA-targeting therapies (Box 1), such as the lack of efficient delivery methods to the central nervous system, undesirable immunogenicity, potential off-target effects, and uncertainties of long-term treatment outcomes. The continuous optimization and development of novel RNA-targeting approaches, including ASOs, siRNAs, small-molecule drugs, as well as viral and non-viral drug delivery methods, will provide safer and more effective options for modulating modifier genes. Beyond the advances in RNA-targeting and delivery methods, identifying the correct gene or pathway to target is particularly challenging for sporadic cases, which comprise most patients. Addressing this requires a deeper and more comprehensive understanding of the basic biology and fundamental molecular mechanisms underlying neurodegenerative phenotypes. The emergence of artificial intelligence (AI) is driving advances in drug design. A number of AI-identified candidate targets have been validated in ALS animal models (Pun et al, 2022; Zhang et al, 2022) and AD models (Merchant et al, 2023). Integrating interdisciplinary novel technologies that can address challenges beyond the reach of traditional methods, developing model systems that more accurately mimic human disease progression, utilizing large databases to predict critical pathogenic pathways or differentiate sub-groups of sporadic diseases, and exploring the genome and understudied candidate genes could provide valuable insights into modifying genes or pathways that may serve as potential therapeutic targets. Overall, there is great anticipation surrounding therapeutic approaches targeting RNAs of the candidate genes in treating neurodegenerative diseases.

Box 1 Questions and future directions Here are some key questions that are important to address and prioritize in order to better understand the role of RNA metabolism dysregulation in neurodegenerative diseases.

**Mechanisms linking RNA dysregulation to pathogenesis:**

- How do specific RBPs and their loss of function or aggregation lead to neurotoxicity?
- What are the critical RNA targets that mediate different aspects of neuronal dysfunction and degeneration?

**Cross-talk between RNA metabolism and other pathways:**

- How does RNA dysregulation interact with other cellular dysfunctions, such as protein aggregation, mitochondrial deficits, stress signaling pathways, and neuroinflammation?
- Does RNA dysregulation serve as a primary trigger or amplify downstream pathological cascades?

**Role of aberrant ncRNAs:**

- Is dysregulation of ncRNAs linked to genetic variants in noncoding regions of the genome associated with neurodegenerative diseases?
- How does dysregulation of ncRNAs exacerbate disease phenotypes?

**Selective vulnerability:**

- What mechanisms underlie the aggregation, mislocalization, or dysregulation of RBPs in various neurodegenerative diseases?
- Are there distinct proteinopathy patterns of RBPs across different neuronal subtypes and brain regions, or can the same RBP pathology predominantly lead to varied dysregulation of RNA processing pathways or targets, thereby contributing to cell type-specific vulnerability?
- Are there protective mechanisms in resistant cell populations?

**Non-cell autonomous toxicity:**

- Is there RBP dysfunction in glial cells, including astrocytes, oligodendrocytes and microglia, that contributes to disease progression in neurodegeneration?
- How is RNA metabolism dysregulated in different glial cell types, and how does this contribute to their toxicity to neurons?

**Disease modeling:**

- How can RBP proteinopathy, including aberrant aggregation, cleavage, modifications and mislocalization, be effectively modeled to mimic disease pathology in both in vitro and in vivo systems?
- How can models be developed to study RNA dysfunctions and pathogenic mechanisms for sporadic diseases?

**Biomarkers and therapeutics:**

- Can diagnostic or prognostic biomarkers be developed based on dysregulated RNA transcripts, to guide therapeutic design and precision medicine in neurodegenerative diseases?
- Is targeting a specific RNA substrate sufficient to rescue disease phenotypes, or is restoring the overall function of the RBP or RNA processing pathway required?

## Supplementary information

Peer Review File

## References

[CR1] (2024) 2024 Alzheimer’s disease facts and figures. Alzheimers Dement 20:3708-3821. https://pubmed.ncbi.nlm.nih.gov/38689398/10.1002/alz.13809 PMC11095490 38689398

[CR2] Abramzon YA, Fratta P, Traynor BJ, Chia R (2020) The overlapping genetics of amyotrophic lateral sclerosis and frontotemporal dementia. Front Neurosci 14:42 32116499 10.3389/fnins.2020.00042 PMC7012787

[CR3] Abrishamdar M, Jalali MS, Rashno M (2022) MALAT1 lncRNA and Parkinson’s disease: the role in the pathophysiology and significance for diagnostic and therapeutic approaches. Mol Neurobiol 59:5253–5262 35665903 10.1007/s12035-022-02899-z

[CR4] Agra Almeida Quadros AR, Li Z, Wang X, Ndayambaje IS, Aryal S, Ramesh N, Nolan M, Jayakumar R, Han Y, Stillman H et al (2024) Cryptic splicing of stathmin-2 and UNC13A mRNAs is a pathological hallmark of TDP-43-associated Alzheimer’s disease. Acta Neuropathol 147:9 38175301 10.1007/s00401-023-02655-0 PMC10766724

[CR5] Ahn I, Kang CS, Han J (2023) Where should siRNAs go: applicable organs for siRNA drugs. Exp Mol Med 55:1283–1292 37430086 10.1038/s12276-023-00998-y PMC10393947

[CR6] Akbarian S, Smith MA, Jones EG (1995) Editing for an AMPA receptor subunit RNA in prefrontal cortex and striatum in Alzheimer’s disease, Huntington’s disease and schizophrenia. Brain Res 699:297–304 8616634 10.1016/0006-8993(95)00922-d

[CR7] Alcolea D, Beeri MS, Rojas JC, Gardner RC, Lleo A (2023) Blood biomarkers in neurodegenerative diseases: implications for the clinical neurologist. Neurology 101:172–180 36878698 10.1212/WNL.0000000000207193 PMC10435056

[CR8] Alessandrini F, Wright M, Kurosaki T, Maquat LE, Kiskinis E (2024) ALS-associated TDP-43 dysfunction compromises UPF1-dependent mRNA metabolism pathways including alternative polyadenylation and 3’UTR length. Preprint at https://www.biorxiv.org/content/10.1101/2024.01.31.578311v1.full.pdf

[CR9] An H, Williams NG, Shelkovnikova TA (2018) NEAT1 and paraspeckles in neurodegenerative diseases: a missing lnc found? Noncoding RNA Res 3:243–252 30533572 10.1016/j.ncrna.2018.11.003 PMC6257911

[CR10] Angelbello AJ, Chen JL, Disney MD (2020) Small molecule targeting of RNA structures in neurological disorders. Ann N Y Acad Sci 1471:57–71 30964958 10.1111/nyas.14051 PMC6785366

[CR11] Arnold ES, Ling SC, Huelga SC, Lagier-Tourenne C, Polymenidou M, Ditsworth D, Kordasiewicz HB, McAlonis-Downes M, Platoshyn O, Parone PA et al (2013) ALS-linked TDP-43 mutations produce aberrant RNA splicing and adult-onset motor neuron disease without aggregation or loss of nuclear TDP-43. Proc Natl Acad Sci USA 110:E736–745 23382207 10.1073/pnas.1222809110 PMC3581922

[CR12] Arnold FJ, Cui Y, Michels S, Colwin MR, Stockford C, Ye W, Tam OH, Menon S, Situ WG, Ehsani KCK et al (2024) TDP-43 dysregulation of polyadenylation site selection is a defining feature of RNA misprocessing in ALS/FTD and related disorders. Preprint at https://www.biorxiv.org/content/10.1101/2024.01.22.576709v1.full.pdf 10.1172/JCI182088 PMC12126230 40454469

[CR13] Arseni D, Chen R, Murzin AG, Peak-Chew SY, Garringer HJ, Newell KL, Kametani F, Robinson AC, Vidal R, Ghetti B et al (2023) TDP-43 forms amyloid filaments with a distinct fold in type A FTLD-TDP. Nature 620:898–903 37532939 10.1038/s41586-023-06405-w PMC10447236

[CR14] Arseni D, Nonaka T, Jacobsen MH, Murzin AG, Cracco L, Peak-Chew SY, Garringer HJ, Kawakami I, Suzuki H, Onaya M et al (2024) Heteromeric amyloid filaments of ANXA11 and TDP-43 in FTLD-TDP Type C. Nature 634:662–668 10.1038/s41586-024-08024-5 PMC11485244 39260416

[CR15] Arun G, Aggarwal D, Spector DL (2020) MALAT1 long non-coding RNA: functional implications. Noncoding RNA 6:22 32503170 10.3390/ncrna6020022 PMC7344863

[CR16] Augustin I, Rosenmund C, Sudhof TC, Brose N (1999) Munc13-1 is essential for fusion competence of glutamatergic synaptic vesicles. Nature 400:457–461 10440375 10.1038/22768

[CR17] Aviner R, Lee TT, Masto VB, Li KH, Andino R, Frydman J (2024) Polyglutamine-mediated ribotoxicity disrupts proteostasis and stress responses in Huntington’s disease. Nat Cell Biol 26:892–902 38741019 10.1038/s41556-024-01414-x PMC12288859

[CR18] Ayala YM, Misteli T, Baralle FE (2008) TDP-43 regulates retinoblastoma protein phosphorylation through the repression of cyclin-dependent kinase 6 expression. Proc Natl Acad Sci USA 105:3785–3789 18305152 10.1073/pnas.0800546105 PMC2268791

[CR19] Ayyildiz D, Bergonzoni G, Monziani A, Tripathi T, Doring J, Kerschbamer E, Di Leva F, Pennati E, Donini L, Kovalenko M et al (2023) CAG repeat expansion in the Huntington’s disease gene shapes linear and circular RNAs biogenesis. PLoS Genet 19:e1010988 37831730 10.1371/journal.pgen.1010988 PMC10617732

[CR20] Bai B, Hales CM, Chen PC, Gozal Y, Dammer EB, Fritz JJ, Wang X, Xia Q, Duong DM, Street C et al (2013) U1 small nuclear ribonucleoprotein complex and RNA splicing alterations in Alzheimer’s disease. Proc Natl Acad Sci USA 110:16562–16567 24023061 10.1073/pnas.1310249110 PMC3799305

[CR21] Bajc Cesnik A, Darovic S, Prpar Mihevc S, Stalekar M, Malnar M, Motaln H, Lee YB, Mazej J, Pohleven J, Grosch M et al (2019) Nuclear RNA foci from C9ORF72 expansion mutation form paraspeckle-like bodies. J Cell Sci 132:jcs224303 30745340 10.1242/jcs.224303

[CR22] Baker DE (2017) Eteplirsen. Hosp Pharm 52:302–305 28515510 10.1310/hpj5204-302 PMC5424835

[CR23] Banez-Coronel M, Ranum LPW (2019) Repeat-associated non-AUG (RAN) translation: insights from pathology. Lab Investig 99:929–942 30918326 10.1038/s41374-019-0241-x PMC7219275

[CR24] Barbosa-Morais NL, Irimia M, Pan Q, Xiong HY, Gueroussov S, Lee LJ, Slobodeniuc V, Kutter C, Watt S, Colak R et al (2012) The evolutionary landscape of alternative splicing in vertebrate species. Science 338:1587–1593 23258890 10.1126/science.1230612

[CR25] Barmada SJ, Ju S, Arjun A, Batarse A, Archbold HC, Peisach D, Li X, Zhang Y, Tank EM, Qiu H et al (2015) Amelioration of toxicity in neuronal models of amyotrophic lateral sclerosis by hUPF1. Proc Natl Acad Sci USA 112:7821–7826 26056265 10.1073/pnas.1509744112 PMC4485101

[CR26] Baron DM, Kaushansky LJ, Ward CL, Sama RR, Chian RJ, Boggio KJ, Quaresma AJ, Nickerson JA, Bosco DA (2013) Amyotrophic lateral sclerosis-linked FUS/TLS alters stress granule assembly and dynamics. Mol Neurodegener 8:30 24090136 10.1186/1750-1326-8-30 PMC3766239

[CR27] Battle DJ, Kasim M, Yong J, Lotti F, Lau CK, Mouaikel J, Zhang Z, Han K, Wan L, Dreyfuss G (2006) The SMN complex: an assembly machine for RNPs. Cold Spring Harb Symp Quant Biol 71:313–320 17381311 10.1101/sqb.2006.71.001

[CR28] Baughn MW, Melamed Z, Lopez-Erauskin J, Beccari MS, Ling K, Zuberi A, Presa M, Gonzalo-Gil E, Maimon R, Vazquez-Sanchez S et al (2023) Mechanism of STMN2 cryptic splice-polyadenylation and its correction for TDP-43 proteinopathies. Science 379:1140–1149 36927019 10.1126/science.abq5622 PMC10148063

[CR29] Belur NR, Bustos BI, Lubbe SJ, Mazzulli JR (2024) Nuclear aggregates of NONO/SFPQ and A-to-I-edited RNA in Parkinson’s disease and dementia with Lewy bodies. Neuron 112:2558–2580.e2513 38761794 10.1016/j.neuron.2024.05.003 PMC11309915

[CR30] Bennett CF, Kordasiewicz HB, Cleveland DW (2021) Antisense drugs make sense for neurological diseases. Annu Rev Pharm Toxicol 61:831–852 10.1146/annurev-pharmtox-010919-023738 PMC8682074 33035446

[CR31] Benson MD, Waddington-Cruz M, Berk JL, Polydefkis M, Dyck PJ, Wang AK, Plante-Bordeneuve V, Barroso FA, Merlini G, Obici L et al (2018) Inotersen treatment for patients with hereditary transthyretin amyloidosis. New Engl J Med 379:22–31 29972757 10.1056/NEJMoa1716793 PMC12611561

[CR32] Bernabo P, Tebaldi T, Groen EJN, Lane FM, Perenthaler E, Mattedi F, Newbery HJ, Zhou H, Zuccotti P, Potrich V et al (2017) In vivo translatome profiling in spinal muscular atrophy reveals a role for SMN protein in ribosome biology. Cell Rep 21:953–965 29069603 10.1016/j.celrep.2017.10.010 PMC5668566

[CR33] Blake LA, Watkins L, Liu Y, Inoue T, Wu B (2024) A rapid inducible RNA decay system reveals fast mRNA decay in P-bodies. Nat Commun 15:2720 38548718 10.1038/s41467-024-46943-z PMC10979015

[CR34] Boccaletto P, Stefaniak F, Ray A, Cappannini A, Mukherjee S, Purta E, Kurkowska M, Shirvanizadeh N, Destefanis E, Groza P et al (2022) MODOMICS: a database of RNA modification pathways. 2021 update. Nucleic Acids Res 50:D231–D235 34893873 10.1093/nar/gkab1083 PMC8728126

[CR35] Borchardt EK, Martinez NM, Gilbert WV (2020) Regulation and function of RNA pseudouridylation in human cells. Annu Rev Genet 54:309–336 32870730 10.1146/annurev-genet-112618-043830 PMC8007080

[CR36] Boros FA, Maszlag-Torok R, Vecsei L, Klivenyi P (2020) Increased level of NEAT1 long non-coding RNA is detectable in peripheral blood cells of patients with Parkinson’s disease. Brain Res 1730:146672 31953211 10.1016/j.brainres.2020.146672

[CR37] Boros FA, Vecsei L, Klivenyi P (2021) NEAT1 on the field of Parkinson’s disease: offense, defense, or a player on the bench? J Parkinsons Dis 11:123–138 33325399 10.3233/JPD-202374 PMC7990444

[CR38] Brown AL, Wilkins OG, Keuss MJ, Hill SE, Zanovello M, Lee WC, Bampton A, Lee FCY, Masino L, Qi YA et al (2022) TDP-43 loss and ALS-risk SNPs drive mis-splicing and depletion of UNC13A. Nature 603:131–137 35197628 10.1038/s41586-022-04436-3 PMC8891020

[CR39] Bryce-Smith S, Brown AL, Mehta PR, Mattedi F, Mikheenko A, Barattucci S, Zanovello M, Dattilo D, Yome M, Hill SE et al (2024) TDP-43 loss induces extensive cryptic polyadenylation in ALS/FTD. Preprint at https://www.biorxiv.org/content/10.1101/2024.01.22.576625v1.full.pdf 10.1038/s41593-025-02050-w PMC12586162 41120751

[CR40] Cabili MN, Trapnell C, Goff L, Koziol M, Tazon-Vega B, Regev A, Rinn JL (2011) Integrative annotation of human large intergenic noncoding RNAs reveals global properties and specific subclasses. Genes Dev 25:1915–1927 21890647 10.1101/gad.17446611 PMC3185964

[CR41] Calliari A, Daughrity LM, Albagli EA, Castellanos Otero P, Yue M, Jansen-West K, Islam NN, Caulfield T, Rawlinson B, DeTure M et al (2024) HDGFL2 cryptic proteins report presence of TDP-43 pathology in neurodegenerative diseases. Mol Neurodegener 19:29 38539264 10.1186/s13024-024-00718-8 PMC10967196

[CR42] Carey JL, Guo L (2022) Liquid-liquid phase separation of TDP-43 and FUS in physiology and pathology of neurodegenerative diseases. Front Mol Biosci 9:826719 35187086 10.3389/fmolb.2022.826719 PMC8847598

[CR43] Castro AF, Loureiro JR, Bessa J, Silveira I (2020) Antisense transcription across nucleotide repeat expansions in neurodegenerative and neuromuscular diseases: progress and mysteries. Genes 11:1418 33261024 10.3390/genes11121418 PMC7760973

[CR44] Castro-Hernandez R, Berulava T, Metelova M, Epple R, Pena Centeno T, Richter J, Kaurani L, Pradhan R, Sakib MS, Burkhardt S et al (2023) Conserved reduction of m(6)A RNA modifications during aging and neurodegeneration is linked to changes in synaptic transcripts. Proc Natl Acad Sci USA 120:e2204933120 36812208 10.1073/pnas.2204933120 PMC9992849

[CR45] Celona B, Dollen JV, Vatsavayai SC, Kashima R, Johnson JR, Tang AA, Hata A, Miller BL, Huang EJ, Krogan NJ et al (2017) Suppression of C9orf72 RNA repeat-induced neurotoxicity by the ALS-associated RNA-binding protein Zfp106. eLife 6:e19032 28072389 10.7554/eLife.19032 PMC5283830

[CR46] Chen HH, Eteleeb A, Wang C, Fernandez MV, Budde JP, Bergmann K, Norton J, Wang F, Ebl C, Morris JC et al (2022a) Circular RNA detection identifies circPSEN1 alterations in brain specific to autosomal dominant Alzheimer’s disease. Acta Neuropathol Commun 10:29 35246267 10.1186/s40478-022-01328-5 PMC8895634

[CR47] Chen JJ, Throop MS, Gehrke L, Kuo I, Pal JK, Brodsky M, London IM (1991) Cloning of the cDNA of the heme-regulated eukaryotic initiation factor 2 alpha (eIF-2 alpha) kinase of rabbit reticulocytes: homology to yeast GCN2 protein kinase and human double-stranded-RNA-dependent eIF-2 alpha kinase. Proc Natl Acad Sci USA 88:7729–7733 1679235 10.1073/pnas.88.17.7729 PMC52376

[CR48] Chen PC, Han X, Shaw TI, Fu Y, Sun H, Niu M, Wang Z, Jiao Y, Teubner BJW, Eddins D et al (2022b) Alzheimer’s disease-associated U1 snRNP splicing dysfunction causes neuronal hyperexcitability and cognitive impairment. Nat Aging 2:923–940 36636325 10.1038/s43587-022-00290-0 PMC9833817

[CR49] Chen Q, Deng N, Lu K, Liao Q, Long X, Gou D, Bi F, Zhou J (2021) Elevated plasma miR-133b and miR-221-3p as biomarkers for early Parkinson’s disease. Sci Rep 11:15268 34315950 10.1038/s41598-021-94734-z PMC8316346

[CR50] Chen X, Yu C, Guo M, Zheng X, Ali S, Huang H, Zhang L, Wang S, Huang Y, Qie S et al (2019) Down-regulation of m^6^A mRNA methylation is involved in dopaminergic neuronal death. ACS Chem Neurosci 10:2355–2363 30835997 10.1021/acschemneuro.8b00657

[CR51] Cheng W, Wang S, Mestre AA, Fu C, Makarem A, Xian F, Hayes LR, Lopez-Gonzalez R, Drenner K, Jiang J et al (2018) C9ORF72 GGGGCC repeat-associated non-AUG translation is upregulated by stress through eIF2α phosphorylation. Nat Commun 9:51 29302060 10.1038/s41467-017-02495-z PMC5754368

[CR52] Chou CC, Zhang Y, Umoh ME, Vaughan SW, Lorenzini I, Liu F, Sayegh M, Donlin-Asp PG, Chen YH, Duong DM et al (2018) TDP-43 pathology disrupts nuclear pore complexes and nucleocytoplasmic transport in ALS/FTD. Nat Neurosci 21:228–239 29311743 10.1038/s41593-017-0047-3 PMC5800968

[CR53] Chu D, von der Haar T (2012) The architecture of eukaryotic translation. Nucleic Acids Res 40:10098–10106 22965119 10.1093/nar/gks825 PMC3488257

[CR54] Cole TA, Zhao H, Collier TJ, Sandoval I, Sortwell CE, Steece-Collier K, Daley BF, Booms A, Lipton J, Welch M et al (2021) alpha-Synuclein antisense oligonucleotides as a disease-modifying therapy for Parkinson’s disease. JCI Insight 6:e135633 33682798 10.1172/jci.insight.135633 PMC8021121

[CR55] Collaborators GBDNSD (2024) Global, regional, and national burden of disorders affecting the nervous system, 1990-2021: a systematic analysis for the Global Burden of Disease Study 2021. Lancet Neurol 23:344–381 38493795 10.1016/S1474-4422(24)00038-3 PMC10949203

[CR56] Conlon EG, Lu L, Sharma A, Yamazaki T, Tang T, Shneider NA, Manley JL (2016) The C9ORF72 GGGGCC expansion forms RNA G-quadruplex inclusions and sequesters hnRNP H to disrupt splicing in ALS brains. eLife 5:e17820 27623008 10.7554/eLife.17820 PMC5050020

[CR57] Conlon EG, Manley JL (2017) RNA-binding proteins in neurodegeneration: mechanisms in aggregate. Genes Dev 31:1509–1528 28912172 10.1101/gad.304055.117 PMC5630017

[CR58] Cooper-Knock J, Walsh MJ, Higginbottom A, Robin Highley J, Dickman MJ, Edbauer D, Ince PG, Wharton SB, Wilson SA, Kirby J et al (2014) Sequestration of multiple RNA recognition motif-containing proteins by C9orf72 repeat expansions. Brain 137:2040–2051 24866055 10.1093/brain/awu120 PMC4065024

[CR59] Couthouis J, Hart MP, Erion R, King OD, Diaz Z, Nakaya T, Ibrahim F, Kim HJ, Mojsilovic-Petrovic J, Panossian S et al (2012) Evaluating the role of the FUS/TLS-related gene EWSR1 in amyotrophic lateral sclerosis. Hum Mol Genet 21:2899–2911 22454397 10.1093/hmg/dds116 PMC3373238

[CR60] Coyne AN, Baskerville V, Zaepfel BL, Dickson DW, Rigo F, Bennett F, Lusk CP, Rothstein JD (2021) Nuclear accumulation of CHMP7 initiates nuclear pore complex injury and subsequent TDP-43 dysfunction in sporadic and familial ALS. Sci Transl Med 13:eabe1923 34321318 10.1126/scitranslmed.abe1923 PMC9022198

[CR61] Coyne AN, Zaepfel BL, Hayes L, Fitchman B, Salzberg Y, Luo EC, Bowen K, Trost H, Aigner S, Rigo F et al (2020) G(4)C(2) Repeat RNA initiates a POM121-mediated reduction in specific nucleoporins in C9orf72 ALS/FTD. Neuron 107:1124–1140.e1111 32673563 10.1016/j.neuron.2020.06.027 PMC8077944

[CR62] Cruz A, Verma M, Wolozin B (2019) The pathophysiology of tau and stress granules in disease. Adv Exp Med Biol 1184:359–372 32096049 10.1007/978-981-32-9358-8_26 PMC8265570

[CR63] Cui Y, Arnold FJ, Peng F, Wang D, Li JS, Michels S, Wagner EJ, La Spada AR, Li W (2023) Alternative polyadenylation transcriptome-wide association study identifies APA-linked susceptibility genes in brain disorders. Nat Commun 14:583 36737438 10.1038/s41467-023-36311-8 PMC9898543

[CR64] Culver BP, Savas JN, Park SK, Choi JH, Zheng S, Zeitlin SO, Yates 3rd JR, Tanese N (2012) Proteomic analysis of wild-type and mutant huntingtin-associated proteins in mouse brains identifies unique interactions and involvement in protein synthesis. J Biol Chem 287:21599–21614 22556411 10.1074/jbc.M112.359307 PMC3381125

[CR65] Daar IO, Maquat LE (1988) Premature translation termination mediates triosephosphate isomerase mRNA degradation. Mol Cell Biol 8:802–813 2832737 10.1128/mcb.8.2.802-813.1988 PMC363207

[CR66] D’Angelo MA, Raices M, Panowski SH, Hetzer MW (2009) Age-dependent deterioration of nuclear pore complexes causes a loss of nuclear integrity in postmitotic cells. Cell 136:284–295 19167330 10.1016/j.cell.2008.11.037 PMC2805151

[CR67] Debes C, Papadakis A, Gronke S, Karalay O, Tain LS, Mizi A, Nakamura S, Hahn O, Weigelt C, Josipovic N et al (2023) Ageing-associated changes in transcriptional elongation influence longevity. Nature 616:814–821 37046086 10.1038/s41586-023-05922-y PMC10132977

[CR68] Decker CJ, Parker R (2012) P-bodies and stress granules: possible roles in the control of translation and mRNA degradation. Cold Spring Harb Perspect Biol 4:a012286 22763747 10.1101/cshperspect.a012286 PMC3428773

[CR69] Delaunay S, Helm M, Frye M (2023) RNA modifications in physiology and disease: towards clinical applications. Nat Rev Genet 25:104–122 10.1038/s41576-023-00645-2 37714958

[CR70] deLorimier E, Hinman MN, Copperman J, Datta K, Guenza M, Berglund JA (2017) Pseudouridine modification inhibits muscleblind-like 1 (MBNL1) binding to CCUG repeats and minimally structured RNA through reduced RNA flexibility. J Biol Chem 292:4350–4357 28130447 10.1074/jbc.M116.770768 PMC5354507

[CR71] Dever TE, Feng L, Wek RC, Cigan AM, Donahue TF, Hinnebusch AG (1992) Phosphorylation of initiation factor 2 alpha by protein kinase GCN2 mediates gene-specific translational control of GCN4 in yeast. Cell 68:585–596 1739968 10.1016/0092-8674(92)90193-g

[CR72] Devi L, Ohno M (2014) PERK mediates eIF2alpha phosphorylation responsible for BACE1 elevation, CREB dysfunction and neurodegeneration in a mouse model of Alzheimer’s disease. Neurobiol Aging 35:2272–2281 24889041 10.1016/j.neurobiolaging.2014.04.031 PMC4127890

[CR73] Dhillon S (2020) Viltolarsen: first approval. Drugs 80:1027–1031 32519222 10.1007/s40265-020-01339-3

[CR74] Ding B, Sepehrimanesh M (2021) Nucleocytoplasmic transport: regulatory mechanisms and the implications in neurodegeneration. Int J Mol Sci 22:4165 33920577 10.3390/ijms22084165 PMC8072611

[CR75] Dolinar A, Koritnik B, Glavac D, Ravnik-Glavac M (2019) Circular RNAs as potential blood biomarkers in amyotrophic lateral sclerosis. Mol Neurobiol 56:8052–8062 31175544 10.1007/s12035-019-1627-x PMC6834740

[CR76] Doma MK, Parker R (2006) Endonucleolytic cleavage of eukaryotic mRNAs with stalls in translation elongation. Nature 440:561–564 16554824 10.1038/nature04530 PMC1839849

[CR77] Dominissini D, Moshitch-Moshkovitz S, Schwartz S, Salmon-Divon M, Ungar L, Osenberg S, Cesarkas K, Jacob-Hirsch J, Amariglio N, Kupiec M et al (2012) Topology of the human and mouse m^6^A RNA methylomes revealed by m^6^A-seq. Nature 485:201–206 22575960 10.1038/nature11112

[CR78] Dominissini D, Nachtergaele S, Moshitch-Moshkovitz S, Peer E, Kol N, Ben-Haim MS, Dai Q, Di Segni A, Salmon-Divon M, Clark WC et al (2016) The dynamic N(1)-methyladenosine methylome in eukaryotic messenger RNA. Nature 530:441–446 26863196 10.1038/nature16998 PMC4842015

[CR79] Dong D, Zhang Z, Li Y, Latallo MJ, Wang S, Nelson B, Wu R, Krishnan G, Gao FB, Wu B et al (2024) Poly-GR repeats associated with ALS/FTD gene C9ORF72 impair translation elongation and induce a ribotoxic stress response in neurons. Sci Signal 17:eadl1030 39106320 10.1126/scisignal.adl1030 PMC11466505

[CR80] Dong X, Bai Y, Liao Z, Gritsch D, Liu X, Wang T, Borges-Monroy R, Ehrlich A, Serrano GE, Feany MB et al (2023) Circular RNAs in the human brain are tailored to neuron identity and neuropsychiatric disease. Nat Commun 14:5327 37723137 10.1038/s41467-023-40348-0 PMC10507039

[CR81] Donnelly CJ, Zhang PW, Pham JT, Haeusler AR, Heusler AR, Mistry NA, Vidensky S, Daley EL, Poth EM, Hoover B et al (2013) RNA toxicity from the ALS/FTD C9ORF72 expansion is mitigated by antisense intervention. Neuron 80:415–428 24139042 10.1016/j.neuron.2013.10.015 PMC4098943

[CR82] Duan L, Zaepfel BL, Aksenova V, Dasso M, Rothstein JD, Kalab P, Hayes LR (2022) Nuclear RNA binding regulates TDP-43 nuclear localization and passive nuclear export. Cell Rep 40:111106 35858577 10.1016/j.celrep.2022.111106 PMC9345261

[CR83] Dube U, Del-Aguila JL, Li Z, Budde JP, Jiang S, Hsu S, Ibanez L, Fernandez MV, Farias F, Norton J et al (2019) An atlas of cortical circular RNA expression in Alzheimer disease brains demonstrates clinical and pathological associations. Nat Neurosci 22:1903–1912 31591557 10.1038/s41593-019-0501-5 PMC6858549

[CR84] Eftekharzadeh B, Daigle JG, Kapinos LE, Coyne A, Schiantarelli J, Carlomagno Y, Cook C, Miller SJ, Dujardin S, Amaral AS et al (2018) Tau protein disrupts nucleocytoplasmic transport in Alzheimer’s disease. Neuron 99:925–940.e927 30189209 10.1016/j.neuron.2018.07.039 PMC6240334

[CR85] Errichelli L, Dini Modigliani S, Laneve P, Colantoni A, Legnini I, Capauto D, Rosa A, De Santis R, Scarfo R, Peruzzi G et al (2017) FUS affects circular RNA expression in murine embryonic stem cell-derived motor neurons. Nat Commun 8:14741 28358055 10.1038/ncomms14741 PMC5379105

[CR86] Eshraghi M, Karunadharma PP, Blin J, Shahani N, Ricci EP, Michel A, Urban NT, Galli N, Sharma M, Ramirez-Jarquin UN et al (2021) Mutant Huntingtin stalls ribosomes and represses protein synthesis in a cellular model of Huntington disease. Nat Commun 12:1461 33674575 10.1038/s41467-021-21637-y PMC7935949

[CR87] Fabian MR, Sonenberg N, Filipowicz W (2010) Regulation of mRNA translation and stability by microRNAs. Annu Rev Biochem 79:351–379 20533884 10.1146/annurev-biochem-060308-103103

[CR88] Fan Y, Lv X, Chen Z, Peng Y, Zhang M (2023) m6A methylation: critical roles in aging and neurological diseases. Front Mol Neurosci 16:1102147 36896007 10.3389/fnmol.2023.1102147 PMC9990872

[CR89] Fardaei M, Rogers MT, Thorpe HM, Larkin K, Hamshere MG, Harper PS, Brook JD (2002) Three proteins, MBNL, MBLL and MBXL, co-localize in vivo with nuclear foci of expanded-repeat transcripts in DM1 and DM2 cells. Hum Mol Genet 11:805–814 11929853 10.1093/hmg/11.7.805

[CR90] Farh KK, Grimson A, Jan C, Lewis BP, Johnston WK, Lim LP, Burge CB, Bartel DP (2005) The widespread impact of mammalian MicroRNAs on mRNA repression and evolution. Science 310:1817–1821 16308420 10.1126/science.1121158

[CR91] Fatica A, Bozzoni I (2014) Long non-coding RNAs: new players in cell differentiation and development. Nat Rev Genet 15:7–21 24296535 10.1038/nrg3606

[CR92] Feiler MS, Strobel B, Freischmidt A, Helferich AM, Kappel J, Brewer BM, Li D, Thal DR, Walther P, Ludolph AC et al (2015) TDP-43 is intercellularly transmitted across axon terminals. J Cell Biol 211:897–911 26598621 10.1083/jcb.201504057 PMC4657165

[CR93] Fernandopulle MS, Lippincott-Schwartz J, Ward ME (2021) RNA transport and local translation in neurodevelopmental and neurodegenerative disease. Nat Neurosci 24:622–632 33510479 10.1038/s41593-020-00785-2 PMC8860725

[CR94] Finkel RS, Mercuri E, Darras BT, Connolly AM, Kuntz NL, Kirschner J, Chiriboga CA, Saito K, Servais L, Tizzano E et al (2017) Nusinersen versus Sham control in infantile-onset spinal muscular atrophy. New Engl J Med 377:1723–1732 29091570 10.1056/NEJMoa1702752

[CR95] Fischl H, Neve J, Wang Z, Patel R, Louey A, Tian B, Furger A (2019) hnRNPC regulates cancer-specific alternative cleavage and polyadenylation profiles. Nucleic Acids Res 47:7580–7591 31147722 10.1093/nar/gkz461 PMC6698646

[CR96] Freibaum BD, Lu Y, Lopez-Gonzalez R, Kim NC, Almeida S, Lee KH, Badders N, Valentine M, Miller BL, Wong PC et al (2015) GGGGCC repeat expansion in C9orf72 compromises nucleocytoplasmic transport. Nature 525:129–133 26308899 10.1038/nature14974 PMC4631399

[CR97] Freibaum BD, Taylor JP (2017) The role of dipeptide repeats in C9ORF72-related ALS-FTD. Front Mol Neurosci 10:35 28243191 10.3389/fnmol.2017.00035 PMC5303742

[CR98] Frischmeyer PA, van Hoof A, O’Donnell K, Guerrerio AL, Parker R, Dietz HC (2002) An mRNA surveillance mechanism that eliminates transcripts lacking termination codons. Science 295:2258–2261 11910109 10.1126/science.1067338

[CR99] Gaisler-Salomon I, Kravitz E, Feiler Y, Safran M, Biegon A, Amariglio N, Rechavi G (2014) Hippocampus-specific deficiency in RNA editing of GluA2 in Alzheimer’s disease. Neurobiol Aging 35:1785–1791 24679603 10.1016/j.neurobiolaging.2014.02.018

[CR100] Garneau NL, Wilusz J, Wilusz CJ (2007) The highways and byways of mRNA decay. Nat Rev Mol Cell Biol 8:113–126 17245413 10.1038/nrm2104

[CR101] Gasset-Rosa F, Chillon-Marinas C, Goginashvili A, Atwal RS, Artates JW, Tabet R, Wheeler VC, Bang AG, Cleveland DW, Lagier-Tourenne C (2017) Polyglutamine-expanded Huntingtin exacerbates age-related disruption of nuclear integrity and nucleocytoplasmic transport. Neuron 94:48–57.e44 28384474 10.1016/j.neuron.2017.03.027 PMC5479704

[CR102] Gebert LFR, MacRae IJ (2019) Regulation of microRNA function in animals. Nat Rev Mol Cell Biol 20:21–37 30108335 10.1038/s41580-018-0045-7 PMC6546304

[CR103] Green KM, Glineburg MR, Kearse MG, Flores BN, Linsalata AE, Fedak SJ, Goldstrohm AC, Barmada SJ, Todd PK (2017) RAN translation at C9orf72-associated repeat expansions is selectively enhanced by the integrated stress response. Nat Commun 8:2005 29222490 10.1038/s41467-017-02200-0 PMC5722904

[CR104] Grima JC, Daigle JG, Arbez N, Cunningham KC, Zhang K, Ochaba J, Geater C, Morozko E, Stocksdale J, Glatzer JC et al (2017) Mutant Huntingtin disrupts the nuclear pore complex. Neuron 94:93–107.e106 28384479 10.1016/j.neuron.2017.03.023 PMC5595097

[CR105] Gruner H, Cortes-Lopez M, Cooper DA, Bauer M, Miura P (2016) CircRNA accumulation in the aging mouse brain. Sci Rep 6:38907 27958329 10.1038/srep38907 PMC5153657

[CR106] Gudde A, van Heeringen SJ, de Oude AI, van Kessel IDG, Estabrook J, Wang ET, Wieringa B, Wansink DG (2017) Antisense transcription of the myotonic dystrophy locus yields low-abundant RNAs with and without (CAG)n repeat. RNA Biol 14:1374–1388 28102759 10.1080/15476286.2017.1279787 PMC5711456

[CR107] Gui X, Luo F, Li Y, Zhou H, Qin Z, Liu Z, Gu J, Xie M, Zhao K, Dai B et al (2019) Structural basis for reversible amyloids of hnRNPA1 elucidates their role in stress granule assembly. Nat Commun 10:2006 31043593 10.1038/s41467-019-09902-7 PMC6494871

[CR108] Haeusler AR, Donnelly CJ, Periz G, Simko EA, Shaw PG, Kim MS, Maragakis NJ, Troncoso JC, Pandey A, Sattler R et al (2014) C9orf72 nucleotide repeat structures initiate molecular cascades of disease. Nature 507:195–200 24598541 10.1038/nature13124 PMC4046618

[CR109] Haeusler AR, Donnelly CJ, Rothstein JD (2016) The expanding biology of the C9orf72 nucleotide repeat expansion in neurodegenerative disease. Nat Rev Neurosci 17:383–395 27150398 10.1038/nrn.2016.38 PMC7376590

[CR110] Hallacli E, Kayatekin C, Nazeen S, Wang XH, Sheinkopf Z, Sathyakumar S, Sarkar S, Jiang X, Dong X, Di Maio R et al (2022) The Parkinson’s disease protein alpha-synuclein is a modulator of processing bodies and mRNA stability. Cell 185:2035–2056.e2033 35688132 10.1016/j.cell.2022.05.008 PMC9394447

[CR111] Harding HP, Zhang Y, Ron D (1999) Protein translation and folding are coupled by an endoplasmic-reticulum-resident kinase. Nature 397:271–274 9930704 10.1038/16729

[CR112] Hartmann H, Hornburg D, Czuppa M, Bader J, Michaelsen M, Farny D, Arzberger T, Mann M, Meissner F, Edbauer D (2018) Proteomics and C9orf72 neuropathology identify ribosomes as poly-GR/PR interactors driving toxicity. Life Sci Alliance 1:e201800070 30456350 10.26508/lsa.201800070 PMC6238541

[CR113] Hedaya OM, Venkata Subbaiah KC, Jiang F, Xie LH, Wu J, Khor ES, Zhu M, Mathews DH, Proschel C, Yao P (2023) Secondary structures that regulate mRNA translation provide insights for ASO-mediated modulation of cardiac hypertrophy. Nat Commun 14:6166 37789015 10.1038/s41467-023-41799-1 PMC10547706

[CR114] Heo YA (2020) Golodirsen: first approval. Drugs 80:329–333 32026421 10.1007/s40265-020-01267-2

[CR115] Hideyama T, Yamashita T, Aizawa H, Tsuji S, Kakita A, Takahashi H, Kwak S (2012) Profound downregulation of the RNA editing enzyme ADAR2 in ALS spinal motor neurons. Neurobiol Dis 45:1121–1128 22226999 10.1016/j.nbd.2011.12.033

[CR116] Hsieh YC, Guo C, Yalamanchili HK, Abreha M, Al-Ouran R, Li Y, Dammer EB, Lah JJ, Levey AI, Bennett DA et al (2019) Tau-mediated disruption of the spliceosome triggers cryptic RNA splicing and neurodegeneration in Alzheimer’s disease. Cell Rep 29:301–316.e310 31597093 10.1016/j.celrep.2019.08.104 PMC6919331

[CR117] Hua Y, Sahashi K, Hung G, Rigo F, Passini MA, Bennett CF, Krainer AR (2010) Antisense correction of SMN2 splicing in the CNS rescues necrosis in a type III SMA mouse model. Genes Dev 24:1634–1644 20624852 10.1101/gad.1941310 PMC2912561

[CR118] Hua Y, Vickers TA, Baker BF, Bennett CF, Krainer AR (2007) Enhancement of SMN2 exon 7 inclusion by antisense oligonucleotides targeting the exon. PLoS Biol 5:e73 17355180 10.1371/journal.pbio.0050073 PMC1820610

[CR119] Huang H, Camats-Perna J, Medeiros R, Anggono V, Widagdo J (2020) Altered expression of the m6A methyltransferase METTL3 in Alzheimer’s disease. eNeuro 7 10.1523/ENEURO.0125-20.2020 PMC7540926 32847866

[CR120] Humphrey J, Birsa N, Milioto C, McLaughlin M, Ule AM, Robaldo D, Eberle AB, Krauchi R, Bentham M, Brown AL et al (2020) FUS ALS-causative mutations impair FUS autoregulation and splicing factor networks through intron retention. Nucleic Acids Res 48:6889–6905 32479602 10.1093/nar/gkaa410 PMC7337901

[CR121] Ikeuchi K, Yazaki E, Kudo K, Inada T (2016) Conserved functions of human Pelota in mRNA quality control of nonstop mRNA. FEBS Lett 590:3254–3263 27543824 10.1002/1873-3468.12366

[CR122] Irwin KE, Jasin P, Braunstein KE, Sinha IR, Garret MA, Bowden KD, Chang K, Troncoso JC, Moghekar A, Oh ES et al (2024) A fluid biomarker reveals loss of TDP-43 splicing repression in presymptomatic ALS-FTD. Nat Med 30:382–393 38278991 10.1038/s41591-023-02788-5 PMC10878965

[CR123] Jackson KL, Dayton RD, Orchard EA, Ju S, Ringe D, Petsko GA, Maquat LE, Klein RL (2015) Preservation of forelimb function by UPF1 gene therapy in a rat model of TDP-43-induced motor paralysis. Gene Ther 22:20–28 25354681 10.1038/gt.2014.101 PMC4924570

[CR124] Jellinger KA (2010) Basic mechanisms of neurodegeneration: a critical update. J Cell Mol Med 14:457–487 20070435 10.1111/j.1582-4934.2010.01010.x PMC3823450

[CR125] Jiang J, Zhu Q, Gendron TF, Saberi S, McAlonis-Downes M, Seelman A, Stauffer JE, Jafar-Nejad P, Drenner K, Schulte D et al (2016) Gain of toxicity from ALS/FTD-linked repeat expansions in C9ORF72 is alleviated by antisense oligonucleotides targeting GGGGCC-containing RNAs. Neuron 90:535–550 27112497 10.1016/j.neuron.2016.04.006 PMC4860075

[CR126] Jiang L, Lin W, Zhang C, Ash PEA, Verma M, Kwan J, van Vliet E, Yang Z, Cruz AL, Boudeau S et al (2021) Interaction of tau with HNRNPA2B1 and *N*^*6*^-methyladenosine RNA mediates the progression of tauopathy. Mol Cell 81:4209–4227.e4212 34453888 10.1016/j.molcel.2021.07.038 PMC8541906

[CR127] Jo M, Lee S, Jeon YM, Kim S, Kwon Y, Kim HJ (2020) The role of TDP-43 propagation in neurodegenerative diseases: integrating insights from clinical and experimental studies. Exp Mol Med 52:1652–1662 33051572 10.1038/s12276-020-00513-7 PMC8080625

[CR128] Jovicic A, Mertens J, Boeynaems S, Bogaert E, Chai N, Yamada SB, Paul 3rd JW, Sun S, Herdy JR, Bieri G et al (2015) Modifiers of C9orf72 dipeptide repeat toxicity connect nucleocytoplasmic transport defects to FTD/ALS. Nat Neurosci 18:1226–1229 26308983 10.1038/nn.4085 PMC4552077

[CR129] Ju S, Tardiff DF, Han H, Divya K, Zhong Q, Maquat LE, Bosco DA, Hayward LJ, Brown Jr RH, Lindquist S et al (2011) A yeast model of FUS/TLS-dependent cytotoxicity. PLoS Biol 9:e1001052 21541368 10.1371/journal.pbio.1001052 PMC3082520

[CR130] Junn E, Lee KW, Jeong BS, Chan TW, Im JY, Mouradian MM (2009) Repression of alpha-synuclein expression and toxicity by microRNA-7. Proc Natl Acad Sci USA 106:13052–13057 19628698 10.1073/pnas.0906277106 PMC2722353

[CR131] Jutzi D, Campagne S, Schmidt R, Reber S, Mechtersheimer J, Gypas F, Schweingruber C, Colombo M, von Schroetter C, Loughlin FE et al (2020) Aberrant interaction of FUS with the U1 snRNA provides a molecular mechanism of FUS induced amyotrophic lateral sclerosis. Nat Commun 11:6341 33311468 10.1038/s41467-020-20191-3 PMC7733473

[CR132] Kamelgarn M, Chen J, Kuang L, Jin H, Kasarskis EJ, Zhu H (2018) ALS mutations of FUS suppress protein translation and disrupt the regulation of nonsense-mediated decay. Proc Natl Acad Sci USA 115:E11904–E11913 30455313 10.1073/pnas.1810413115 PMC6304956

[CR133] Kanadia RN, Johnstone KA, Mankodi A, Lungu C, Thornton CA, Esson D, Timmers AM, Hauswirth WW, Swanson MS (2003) A muscleblind knockout model for myotonic dystrophy. Science 302:1978–1980 14671308 10.1126/science.1088583

[CR134] Kanadia RN, Shin J, Yuan Y, Beattie SG, Wheeler TM, Thornton CA, Swanson MS (2006) Reversal of RNA missplicing and myotonia after muscleblind overexpression in a mouse poly(CUG) model for myotonic dystrophy. Proc Natl Acad Sci USA 103:11748–11753 16864772 10.1073/pnas.0604970103 PMC1544241

[CR135] Kanekura K, Yagi T, Cammack AJ, Mahadevan J, Kuroda M, Harms MB, Miller TM, Urano F (2016) Poly-dipeptides encoded by the C9ORF72 repeats block global protein translation. Hum Mol Genet 25:1803–1813 26931465 10.1093/hmg/ddw052 PMC4986334

[CR136] Kawahara Y, Ito K, Sun H, Aizawa H, Kanazawa I, Kwak S (2004) Glutamate receptors: RNA editing and death of motor neurons. Nature 427:801 14985749 10.1038/427801a

[CR137] Kawahara Y, Kwak S, Sun H, Ito K, Hashida H, Aizawa H, Jeong SY, Kanazawa I (2003) Human spinal motoneurons express low relative abundance of GluR2 mRNA: an implication for excitotoxicity in ALS. J Neurochem 85:680–689 12694394 10.1046/j.1471-4159.2003.01703.x

[CR138] Keuss MJ, Harley P, Ryadnov E, Jackson RE, Zanovello M, Wilkins OG, Barattucci S, Mehta PR, Oliveira MG, Parkes JE et al (2024) Loss of TDP-43 induces synaptic dysfunction that is rescued by *UNC13A* splice-switching ASOs. Preprint at https://www.biorxiv.org/content/10.1101/2024.06.20.599684v1.full.pdf

[CR139] Khalil B, Chhangani D, Wren MC, Smith CL, Lee JH, Li X, Puttinger C, Tsai CW, Fortin G, Morderer D et al (2022) Nuclear import receptors are recruited by FG-nucleoporins to rescue hallmarks of TDP-43 proteinopathy. Mol Neurodegener 17:80 36482422 10.1186/s13024-022-00585-1 PMC9733332

[CR140] Khan AU, Qu R, Ouyang J, Dai J (2020) Role of nucleoporins and transport receptors in cell differentiation. Front Physiol 11:239 32308628 10.3389/fphys.2020.00239 PMC7145948

[CR141] Khermesh K, D’Erchia AM, Barak M, Annese A, Wachtel C, Levanon EY, Picardi E, Eisenberg E (2016) Reduced levels of protein recoding by A-to-I RNA editing in Alzheimer’s disease. RNA 22:290–302 26655226 10.1261/rna.054627.115 PMC4712678

[CR142] Khoddami V, Yerra A, Mosbruger TL, Fleming AM, Burrows CJ, Cairns BR (2019) Transcriptome-wide profiling of multiple RNA modifications simultaneously at single-base resolution. Proc Natl Acad Sci USA 116:6784–6789 30872485 10.1073/pnas.1817334116 PMC6452723

[CR143] Khorkova O, Stahl J, Joji A, Volmar CH, Wahlestedt C (2023) Amplifying gene expression with RNA-targeted therapeutics. Nat Rev Drug Discov 22:539–561 37253858 10.1038/s41573-023-00704-7 PMC10227815

[CR144] Khosravi B, Hartmann H, May S, Mohl C, Ederle H, Michaelsen M, Schludi MH, Dormann D, Edbauer D (2017) Cytoplasmic poly-GA aggregates impair nuclear import of TDP-43 in C9orf72 ALS/FTLD. Hum Mol Genet 26:790–800 28040728 10.1093/hmg/ddw432 PMC5409121

[CR145] Kim E, Kim YK, Lee SV (2021) Emerging functions of circular RNA in aging. Trends Genet 37:819–829 34016449 10.1016/j.tig.2021.04.014

[CR146] Kim HJ, Kim NC, Wang YD, Scarborough EA, Moore J, Diaz Z, MacLea KS, Freibaum B, Li S, Molliex A et al (2013) Mutations in prion-like domains in hnRNPA2B1 and hnRNPA1 cause multisystem proteinopathy and ALS. Nature 495:467–473 23455423 10.1038/nature11922 PMC3756911

[CR147] Kim J, Hu C, Moufawad El Achkar C, Black LE, Douville J, Larson A, Pendergast MK, Goldkind SF, Lee EA, Kuniholm A et al (2019) Patient-customized oligonucleotide therapy for a rare genetic disease. New Engl J Med 381:1644–1652 31597037 10.1056/NEJMoa1813279 PMC6961983

[CR148] Kim J, Inoue K, Ishii J, Vanti WB, Voronov SV, Murchison E, Hannon G, Abeliovich A (2007) A MicroRNA feedback circuit in midbrain dopamine neurons. Science 317:1220–1224 17761882 10.1126/science.1140481 PMC2782470

[CR149] Kim JE, Hong YH, Kim JY, Jeon GS, Jung JH, Yoon BN, Son SY, Lee KW, Kim JI, Sung JJ (2017) Altered nucleocytoplasmic proteome and transcriptome distributions in an in vitro model of amyotrophic lateral sclerosis. PLoS ONE 12:e0176462 28453527 10.1371/journal.pone.0176462 PMC5409181

[CR150] Kim YE, Hosp F, Frottin F, Ge H, Mann M, Hayer-Hartl M, Hartl FU (2016) Soluble oligomers of PolyQ-expanded huntingtin target a multiplicity of key cellular factors. Mol Cell 63:951–964 27570076 10.1016/j.molcel.2016.07.022

[CR151] Kingwell K (2021) Double setback for ASO trials in Huntington disease. Nat Rev Drug Discov 20:412–413 34012000 10.1038/d41573-021-00088-6

[CR152] Klebe C, Bischoff FR, Ponstingl H, Wittinghofer A (1995) Interaction of the nuclear GTP-binding protein Ran with its regulatory proteins RCC1 and RanGAP1. Biochemistry 34:639–647 7819259 10.1021/bi00002a031

[CR153] Klim JR, Williams LA, Limone F, Guerra San Juan I, Davis-Dusenbery BN, Mordes DA, Burberry A, Steinbaugh MJ, Gamage KK, Kirchner R et al (2019) ALS-implicated protein TDP-43 sustains levels of STMN2, a mediator of motor neuron growth and repair. Nat Neurosci 22:167–179 30643292 10.1038/s41593-018-0300-4 PMC7153761

[CR154] Kocerha J, Xu Y, Prucha MS, Zhao D, Chan AW (2014) microRNA-128a dysregulation in transgenic Huntington’s disease monkeys. Mol Brain 7:46 24929669 10.1186/1756-6606-7-46 PMC4065582

[CR155] Kordasiewicz HB, Stanek LM, Wancewicz EV, Mazur C, McAlonis MM, Pytel KA, Artates JW, Weiss A, Cheng SH, Shihabuddin LS et al (2012) Sustained therapeutic reversal of Huntington’s disease by transient repression of huntingtin synthesis. Neuron 74:1031–1044 22726834 10.1016/j.neuron.2012.05.009 PMC3383626

[CR156] Kraus TFJ, Haider M, Spanner J, Steinmaurer M, Dietinger V, Kretzschmar HA (2017) Altered long noncoding RNA expression precedes the course of Parkinson’s disease—a preliminary report. Mol Neurobiol 54:2869–2877 27021022 10.1007/s12035-016-9854-x

[CR157] Krus KL, Strickland A, Yamada Y, Devault L, Schmidt RE, Bloom AJ, Milbrandt J, DiAntonio A (2022) Loss of Stathmin-2, a hallmark of TDP-43-associated ALS, causes motor neuropathy. Cell Rep 39:111001 35767949 10.1016/j.celrep.2022.111001 PMC9327139

[CR158] Kwiatkowski Jr. TJ, Bosco DA, Leclerc AL, Tamrazian E, Vanderburg CR, Russ C, Davis A, Gilchrist J, Kasarskis EJ, Munsat T et al (2009) Mutations in the FUS/TLS gene on chromosome 16 cause familial amyotrophic lateral sclerosis. Science 323:1205–1208 19251627 10.1126/science.1166066

[CR159] Kwon D (2021) Failure of genetic therapies for Huntington’s devastates community. Nature 593:180 33963316 10.1038/d41586-021-01177-7

[CR160] Lagier-Tourenne C, Polymenidou M, Cleveland DW (2010) TDP-43 and FUS/TLS: emerging roles in RNA processing and neurodegeneration. Hum Mol Genet 19:R46–64 20400460 10.1093/hmg/ddq137 PMC3167692

[CR161] Lang Y, Li Y, Yu H, Lin L, Chen X, Wang S, Zhang H (2020) HOTAIR drives autophagy in midbrain dopaminergic neurons in the substantia nigra compacta in a mouse model of Parkinson’s disease by elevating NPTX2 via miR-221-3p binding. Aging 12:7660–7678 32396526 10.18632/aging.103028 PMC7244061

[CR162] Lasda E, Parker R (2014) Circular RNAs: diversity of form and function. RNA 20:1829–1842 25404635 10.1261/rna.047126.114 PMC4238349

[CR163] Latallo MJ, Wang S, Dong D, Nelson B, Livingston NM, Wu R, Zhao N, Stasevich TJ, Bassik MC, Sun S et al (2023) Single-molecule imaging reveals distinct elongation and frameshifting dynamics between frames of expanded RNA repeats in C9ORF72-ALS/FTD. Nat Commun 14:5581 37696852 10.1038/s41467-023-41339-x PMC10495369

[CR164] Lauria F, Bernabo P, Tebaldi T, Groen EJN, Perenthaler E, Maniscalco F, Rossi A, Donzel D, Clamer M, Marchioretto M et al (2020) SMN-primed ribosomes modulate the translation of transcripts related to spinal muscular atrophy. Nat Cell Biol 22:1239–1251 32958857 10.1038/s41556-020-00577-7 PMC7610479

[CR165] Lee HG, Ueda M, Miyamoto Y, Yoneda Y, Perry G, Smith MA, Zhu X (2006) Aberrant localization of importin alpha1 in hippocampal neurons in Alzheimer disease. Brain Res 1124:1–4 17070506 10.1016/j.brainres.2006.09.084

[CR166] Lee ST, Chu K, Im WS, Yoon HJ, Im JY, Park JE, Park KH, Jung KH, Lee SK, Kim M et al (2011) Altered microRNA regulation in Huntington’s disease models. Exp Neurol 227:172–179 21035445 10.1016/j.expneurol.2010.10.012

[CR167] Leeds P, Peltz SW, Jacobson A, Culbertson MR (1991) The product of the yeast UPF1 gene is required for rapid turnover of mRNAs containing a premature translational termination codon. Genes Dev 5:2303–2314 1748286 10.1101/gad.5.12a.2303

[CR168] Lefebvre S, Burglen L, Reboullet S, Clermont O, Burlet P, Viollet L, Benichou B, Cruaud C, Millasseau P, Zeviani M et al (1995) Identification and characterization of a spinal muscular atrophy-determining gene. Cell 80:155–165 7813012 10.1016/0092-8674(95)90460-3

[CR169] Lester E, Ooi FK, Bakkar N, Ayers J, Woerman AL, Wheeler J, Bowser R, Carlson GA, Prusiner SB, Parker R (2021) Tau aggregates are RNA-protein assemblies that mislocalize multiple nuclear speckle components. Neuron 109:1675–1691.e1679 33848474 10.1016/j.neuron.2021.03.026 PMC8141031

[CR170] Lester E, Parker R (2018) The Tau of nuclear-cytoplasmic transport. Neuron 99:869–871 30189205 10.1016/j.neuron.2018.08.026

[CR171] Levin AA (2019) Treating disease at the RNA level with oligonucleotides. New Engl J Med 380:57–70 30601736 10.1056/NEJMra1705346

[CR172] Li D, McIntosh CS, Mastaglia FL, Wilton SD, Aung-Htut MT (2021) Neurodegenerative diseases: a hotbed for splicing defects and the potential therapies. Transl Neurodegener 10:16 34016162 10.1186/s40035-021-00240-7 PMC8136212

[CR173] Li Q, Gloudemans MJ, Geisinger JM, Fan B, Aguet F, Sun T, Ramaswami G, Li YI, Ma JB, Pritchard JK et al (2022) RNA editing underlies genetic risk of common inflammatory diseases. Nature 608:569–577 35922514 10.1038/s41586-022-05052-x PMC9790998

[CR174] Li S, Lv X, Zhai K, Xu R, Zhang Y, Zhao S, Qin X, Yin L, Lou J (2016) MicroRNA-7 inhibits neuronal apoptosis in a cellular Parkinson’s disease model by targeting Bax and Sirt2. Am J Transl Res 8:993–1004 27158385 PMC4846942

[CR175] Li X, Yang L, Chen LL (2018) The biogenesis, functions, and challenges of circular RNAs. Mol Cell 71:428–442 30057200 10.1016/j.molcel.2018.06.034

[CR176] Li Y, Dou X, Liu J, Xiao Y, Zhang Z, Hayes L, Wu R, Fu X, Ye Y, Yang B et al (2023) Globally reduced N(6)-methyladenosine (m(6)A) in C9ORF72-ALS/FTD dysregulates RNA metabolism and contributes to neurodegeneration. Nat Neurosci 26:1328–1338 37365312 10.1038/s41593-023-01374-9 PMC11361766

[CR177] Li YR, King OD, Shorter J, Gitler AD (2013) Stress granules as crucibles of ALS pathogenesis. J Cell Biol 201:361–372 23629963 10.1083/jcb.201302044 PMC3639398

[CR178] Liang D, Tatomer DC, Luo Z, Wu H, Yang L, Chen LL, Cherry S, Wilusz JE (2017a) The output of protein-coding genes shifts to circular RNAs when the pre-mRNA processing machinery is limiting. Mol Cell 68:940–954.e943 29174924 10.1016/j.molcel.2017.10.034 PMC5728686

[CR179] Liang XH, Sun H, Shen W, Wang S, Yao J, Migawa MT, Bui HH, Damle SS, Riney S, Graham MJ et al (2017b) Antisense oligonucleotides targeting translation inhibitory elements in 5’ UTRs can selectively increase protein levels. Nucleic Acids Res 45:9528–9546 28934489 10.1093/nar/gkx632 PMC5766168

[CR180] Lima JF, Cerqueira L, Figueiredo C, Oliveira C, Azevedo NF (2018) Anti-miRNA oligonucleotides: a comprehensive guide for design. RNA Biol 15:338–352 29570036 10.1080/15476286.2018.1445959 PMC5927725

[CR181] Lin X, Miller JW, Mankodi A, Kanadia RN, Yuan Y, Moxley RT, Swanson MS, Thornton CA (2006) Failure of MBNL1-dependent post-natal splicing transitions in myotonic dystrophy. Hum Mol Genet 15:2087–2097 16717059 10.1093/hmg/ddl132

[CR182] Lindberg MJ, Bystrom R, Boknas N, Andersen PM, Oliveberg M (2005) Systematically perturbed folding patterns of amyotrophic lateral sclerosis (ALS)-associated SOD1 mutants. Proc Natl Acad Sci USA 102:9754–9759 15987780 10.1073/pnas.0501957102 PMC1174986

[CR183] Ling JP, Pletnikova O, Troncoso JC, Wong PC (2015) TDP-43 repression of nonconserved cryptic exons is compromised in ALS-FTD. Science 349:650–655 26250685 10.1126/science.aab0983 PMC4825810

[CR184] Ling SC, Polymenidou M, Cleveland DW (2013) Converging mechanisms in ALS and FTD: disrupted RNA and protein homeostasis. Neuron 79:416–438 23931993 10.1016/j.neuron.2013.07.033 PMC4411085

[CR185] Liu EY, Cali CP, Lee EB (2017a) RNA metabolism in neurodegenerative disease. Dis Model Mech 10:509–518 28468937 10.1242/dmm.028613 PMC5451173

[CR186] Liu SJ, Horlbeck MA, Cho SW, Birk HS, Malatesta M, He D, Attenello FJ, Villalta JE, Cho MY, Chen Y et al (2017b) CRISPRi-based genome-scale identification of functional long noncoding RNA loci in human cells. Science 355:aah7111 27980086 10.1126/science.aah7111 PMC5394926

[CR187] Liu Y, Andreucci A, Iwamoto N, Yin Y, Yang H, Liu F, Bulychev A, Hu XS, Lin X, Lamore S et al (2022) Preclinical evaluation of WVE-004, aninvestigational stereopure oligonucleotide for the treatment of C9orf72-associated ALS or FTD. Mol Ther Nucleic Acids 28:558–570 35592494 10.1016/j.omtn.2022.04.007 PMC9092894

[CR188] Liu Y, Lu Z (2018) Long non-coding RNA NEAT1 mediates the toxic of Parkinson’s disease induced by MPTP/MPP+ via regulation of gene expression. Clin Exp Pharm Physiol 45:841–848 10.1111/1440-1681.12932 29575151

[CR189] Liu-Yesucevitz L, Bilgutay A, Zhang YJ, Vanderweyde T, Citro A, Mehta T, Zaarur N, McKee A, Bowser R, Sherman M et al (2010) Tar DNA binding protein-43 (TDP-43) associates with stress granules: analysis of cultured cells and pathological brain tissue. PLoS ONE 5:e13250 20948999 10.1371/journal.pone.0013250 PMC2952586

[CR190] Livneh I, Moshitch-Moshkovitz S, Amariglio N, Rechavi G, Dominissini D (2020) The m^6^A epitranscriptome: transcriptome plasticity in brain development and function. Nat Rev Neurosci 21:36–51 31804615 10.1038/s41583-019-0244-z

[CR191] Loeffler T, Schilcher I, Flunkert S, Hutter-Paier B (2020) Neurofilament-light chain as biomarker of neurodegenerative and rare diseases with high translational value. Front Neurosci 14:579 32595447 10.3389/fnins.2020.00579 PMC7300175

[CR192] Lopez-Erauskin J, Bravo-Hernandez M, Presa M, Baughn MW, Melamed Z, Beccari MS, Agra de Almeida Quadros AR, Arnold-Garcia O, Zuberi A, Ling K et al (2024) Stathmin-2 loss leads to neurofilament-dependent axonal collapse driving motor and sensory denervation. Nat Neurosci 27:34–47 37996528 10.1038/s41593-023-01496-0 PMC10842032

[CR193] Lopez-Erauskin J, Tadokoro T, Baughn MW, Myers B, McAlonis-Downes M, Chillon-Marinas C, Asiaban JN, Artates J, Bui AT, Vetto AP et al (2018) ALS/FTD-linked mutation in FUS suppresses intra-axonal protein synthesis and drives disease without nuclear loss-of-function of FUS. Neuron 100:816–830.e817 30344044 10.1016/j.neuron.2018.09.044 PMC6277851

[CR194] Lotti F, Imlach WL, Saieva L, Beck ES, Hao le T, Li DK, Jiao W, Mentis GZ, Beattie CE, McCabe BD et al (2012) An SMN-dependent U12 splicing event essential for motor circuit function. Cell 151:440–454 23063131 10.1016/j.cell.2012.09.012 PMC3474596

[CR195] Lourenco MV, Clarke JR, Frozza RL, Bomfim TR, Forny-Germano L, Batista AF, Sathler LB, Brito-Moreira J, Amaral OB, Silva CA et al (2013) TNF-alpha mediates PKR-dependent memory impairment and brain IRS-1 inhibition induced by Alzheimer’s beta-amyloid oligomers in mice and monkeys. Cell Metab 18:831–843 24315369 10.1016/j.cmet.2013.11.002

[CR196] Loveland AB, Svidritskiy E, Susorov D, Lee S, Park A, Zvornicanin S, Demo G, Gao FB, Korostelev AA (2022) Ribosome inhibition by C9ORF72-ALS/FTD-associated poly-PR and poly-GR proteins revealed by cryo-EM. Nat Commun 13:2776 35589706 10.1038/s41467-022-30418-0 PMC9120013

[CR197] Lu YN, Kavianpour S, Zhang T, Zhang X, Nguyen D, Thombre R, He L, Wang J (2021) MARK2 phosphorylates eIF2alpha in response to proteotoxic stress. PLoS Biol 19:e3001096 33705388 10.1371/journal.pbio.3001096 PMC7951919

[CR198] Luisier R, Tyzack GE, Hall CE, Mitchell JS, Devine H, Taha DM, Malik B, Meyer I, Greensmith L, Newcombe J et al (2018) Intron retention and nuclear loss of SFPQ are molecular hallmarks of ALS. Nat Commun 9:2010 29789581 10.1038/s41467-018-04373-8 PMC5964114

[CR199] Lyon AS, Peeples WB, Rosen MK (2021) A framework for understanding the functions of biomolecular condensates across scales. Nat Rev Mol Cell Biol 22:215–235 33169001 10.1038/s41580-020-00303-z PMC8574987

[CR200] Ma T, Trinh MA, Wexler AJ, Bourbon C, Gatti E, Pierre P, Cavener DR, Klann E (2013) Suppression of eIF2alpha kinases alleviates Alzheimer’s disease-related plasticity and memory deficits. Nat Neurosci 16:1299–1305 23933749 10.1038/nn.3486 PMC3756900

[CR201] Ma XR, Prudencio M, Koike Y, Vatsavayai SC, Kim G, Harbinski F, Briner A, Rodriguez CM, Guo C, Akiyama T et al (2022) TDP-43 represses cryptic exon inclusion in the FTD-ALS gene UNC13A. Nature 603:124–130 35197626 10.1038/s41586-022-04424-7 PMC8891019

[CR202] Mackenzie IR, Nicholson AM, Sarkar M, Messing J, Purice MD, Pottier C, Annu K, Baker M, Perkerson RB, Kurti A et al (2017) TIA1 mutations in amyotrophic lateral sclerosis and frontotemporal dementia promote phase separation and alter stress granule dynamics. Neuron 95:808–816.e809 28817800 10.1016/j.neuron.2017.07.025 PMC5576574

[CR203] Mackenzie IR, Rademakers R, Neumann M (2010) TDP-43 and FUS in amyotrophic lateral sclerosis and frontotemporal dementia. Lancet Neurol 9:995–1007 20864052 10.1016/S1474-4422(10)70195-2

[CR204] Mankodi A, Urbinati CR, Yuan QP, Moxley RT, Sansone V, Krym M, Henderson D, Schalling M, Swanson MS, Thornton CA (2001) Muscleblind localizes to nuclear foci of aberrant RNA in myotonic dystrophy types 1 and 2. Hum Mol Genet 10:2165–2170 11590133 10.1093/hmg/10.19.2165

[CR205] Mann JR, Gleixner AM, Mauna JC, Gomes E, DeChellis-Marks MR, Needham PG, Copley KE, Hurtle B, Portz B, Pyles NJ et al (2019) RNA binding antagonizes neurotoxic phase transitions of TDP-43. Neuron 102:321–338.e328 30826182 10.1016/j.neuron.2019.01.048 PMC6472983

[CR206] Maquat LE (2004) Nonsense-mediated mRNA decay: splicing, translation and mRNP dynamics. Nat Rev Mol Cell Biol 5:89–99 15040442 10.1038/nrm1310

[CR207] Marcelo A, Koppenol R, de Almeida LP, Matos CA, Nobrega C (2021) Stress granules, RNA-binding proteins and polyglutamine diseases: too much aggregation? Cell Death Dis 12:592 34103467 10.1038/s41419-021-03873-8 PMC8187637

[CR208] Markmiller S, Sathe S, Server KL, Nguyen TB, Fulzele A, Cody N, Javaherian A, Broski S, Finkbeiner S, Bennett EJ et al (2021) Persistent mRNA localization defects and cell death in ALS neurons caused by transient cellular stress. Cell Rep 36:109685 34496257 10.1016/j.celrep.2021.109685 PMC11341010

[CR209] Marti E, Pantano L, Banez-Coronel M, Llorens F, Minones-Moyano E, Porta S, Sumoy L, Ferrer I, Estivill X (2010) A myriad of miRNA variants in control and Huntington’s disease brain regions detected by massively parallel sequencing. Nucleic Acids Res 38:7219–7235 20591823 10.1093/nar/gkq575 PMC2978354

[CR210] Martin LJ, Adams DA, Niedzwiecki MV, Wong M (2022) Aberrant DNA and RNA methylation occur in spinal cord and skeletal muscle of human SOD1 mouse models of ALS and in human ALS: targeting DNA methylation is therapeutic. Cells 11:3448 36359844 10.3390/cells11213448 PMC9657572

[CR211] McDonald NA, Fetter RD, Shen K (2020) Assembly of synaptic active zones requires phase separation of scaffold molecules. Nature 588:454–458 33208945 10.1038/s41586-020-2942-0

[CR212] McEachin ZT, Gendron TF, Raj N, Garcia-Murias M, Banerjee A, Purcell RH, Ward PJ, Todd TW, Merritt-Garza ME, Jansen-West K et al (2020) Chimeric peptide species contribute to divergent dipeptide repeat pathology in c9ALS/FTD and SCA36. Neuron 107:292–305.e296 32375063 10.1016/j.neuron.2020.04.011 PMC8138626

[CR213] McKeever PM, Sababi AM, Sharma R, Khuu N, Xu Z, Shen SY, Xiao S, McGoldrick P, Orouji E, Ketela T et al (2023) Single-nucleus multiomic atlas of frontal cortex in amyotrophic lateral sclerosis with a deep learning-based decoding of alternative polyadenylation mechanisms. Preprint at https://www.biorxiv.org/content/10.1101/2023.12.22.573083v1

[CR214] McMillan KJ, Murray TK, Bengoa-Vergniory N, Cordero-Llana O, Cooper J, Buckley A, Wade-Martins R, Uney JB, O’Neill MJ, Wong LF et al (2017) Loss of microRNA-7 regulation leads to alpha-synuclein accumulation and dopaminergic neuronal loss in vivo. Mol Ther 25:2404–2414 28927576 10.1016/j.ymthe.2017.08.017 PMC5628933

[CR215] McMillan M, Gomez N, Hsieh C, Bekier M, Li X, Miguez R, Tank EMH, Barmada SJ (2023) RNA methylation influences TDP43 binding and disease pathogenesis in models of amyotrophic lateral sclerosis and frontotemporal dementia. Mol Cell 83:219–236.e217 36634675 10.1016/j.molcel.2022.12.019 PMC9899051

[CR216] McNeill E, Van Vactor D (2012) MicroRNAs shape the neuronal landscape. Neuron 75:363–379 22884321 10.1016/j.neuron.2012.07.005 PMC3441179

[CR217] Melamed Z, Lopez-Erauskin J, Baughn MW, Zhang O, Drenner K, Sun Y, Freyermuth F, McMahon MA, Beccari MS, Artates JW et al (2019) Premature polyadenylation-mediated loss of stathmin-2 is a hallmark of TDP-43-dependent neurodegeneration. Nat Neurosci 22:180–190 30643298 10.1038/s41593-018-0293-z PMC6348009

[CR218] Meneses A, Koga S, O’Leary J, Dickson DW, Bu G, Zhao N (2021) TDP-43 pathology in Alzheimer’s disease. Mol Neurodegener 16:84 34930382 10.1186/s13024-021-00503-x PMC8691026

[CR219] Meng S, Zhou H, Feng Z, Xu Z, Tang Y, Li P, Wu M (2017) CircRNA: functions and properties of a novel potential biomarker for cancer. Mol Cancer 16:94 28535767 10.1186/s12943-017-0663-2 PMC5440908

[CR220] Merchant JP, Zhu K, Henrion MYR, Zaidi SSA, Lau B, Moein S, Alamprese ML, Pearse 2nd RV, Bennett DA, Ertekin-Taner N et al (2023) Predictive network analysis identifies JMJD6 and other potential key drivers in Alzheimer’s disease. Commun Biol 6:503 37188718 10.1038/s42003-023-04791-5 PMC10185548

[CR221] Merkin J, Russell C, Chen P, Burge CB (2012) Evolutionary dynamics of gene and isoform regulation in mammalian tissues. Science 338:1593–1599 23258891 10.1126/science.1228186 PMC3568499

[CR222] Miller JW, Urbinati CR, Teng-Umnuay P, Stenberg MG, Byrne BJ, Thornton CA, Swanson MS (2000) Recruitment of human muscleblind proteins to (CUG)(n) expansions associated with myotonic dystrophy. EMBO J 19:4439–4448 10970838 10.1093/emboj/19.17.4439 PMC302046

[CR223] Miller TM, Cudkowicz ME, Genge A, Shaw PJ, Sobue G, Bucelli RC, Chio A, Van Damme P, Ludolph AC, Glass JD et al (2022) Trial of antisense oligonucleotide tofersen for SOD1 ALS. New Engl J Med 387:1099–1110 36129998 10.1056/NEJMoa2204705

[CR224] Mirra A, Rossi S, Scaricamazza S, Di Salvio M, Salvatori I, Valle C, Rusmini P, Poletti A, Cestra G, Carri MT et al (2017) Functional interaction between FUS and SMN underlies SMA-like splicing changes in wild-type hFUS mice. Sci Rep 7:2033 28515487 10.1038/s41598-017-02195-0 PMC5435706

[CR225] Moens TG, Niccoli T, Wilson KM, Atilano ML, Birsa N, Gittings LM, Holbling BV, Dyson MC, Thoeng A, Neeves J et al (2019) C9orf72 arginine-rich dipeptide proteins interact with ribosomal proteins in vivo to induce a toxic translational arrest that is rescued by eIF1A. Acta Neuropathol 137:487–500 30604225 10.1007/s00401-018-1946-4 PMC6514073

[CR226] Molliex A, Temirov J, Lee J, Coughlin M, Kanagaraj AP, Kim HJ, Mittag T, Taylor JP (2015) Phase separation by low complexity domains promotes stress granule assembly and drives pathological fibrillization. Cell 163:123–133 26406374 10.1016/j.cell.2015.09.015 PMC5149108

[CR227] Moore S, Alsop E, Lorenzini I, Starr A, Rabichow BE, Mendez E, Levy JL, Burciu C, Reiman R, Chew J et al (2019) ADAR2 mislocalization and widespread RNA editing aberrations in C9orf72-mediated ALS/FTD. Acta Neuropathol 138:49–65 30945056 10.1007/s00401-019-01999-w PMC6750285

[CR228] Mori K, Lammich S, Mackenzie IR, Forne I, Zilow S, Kretzschmar H, Edbauer D, Janssens J, Kleinberger G, Cruts M et al (2013) hnRNP A3 binds to GGGGCC repeats and is a constituent of p62-positive/TDP43-negative inclusions in the hippocampus of patients with C9orf72 mutations. Acta Neuropathol 125:413–423 23381195 10.1007/s00401-013-1088-7

[CR229] Murthy AC, Dignon GL, Kan Y, Zerze GH, Parekh SH, Mittal J, Fawzi NL (2019) Molecular interactions underlying liquid-liquid phase separation of the FUS low-complexity domain. Nat Struct Mol Biol 26:637–648 31270472 10.1038/s41594-019-0250-x PMC6613800

[CR230] Nachury MV, Weis K (1999) The direction of transport through the nuclear pore can be inverted. Proc Natl Acad Sci USA 96:9622–9627 10449743 10.1073/pnas.96.17.9622 PMC22259

[CR231] Neil EE, Bisaccia EK (2019) Nusinersen: a novel antisense oligonucleotide for the treatment of spinal muscular atrophy. J Pediatr Pharm Ther 24:194–203 10.5863/1551-6776-24.3.194 PMC6510522 31093018

[CR232] Neumann M, Bentmann E, Dormann D, Jawaid A, DeJesus-Hernandez M, Ansorge O, Roeber S, Kretzschmar HA, Munoz DG, Kusaka H et al (2011) FET proteins TAF15 and EWS are selective markers that distinguish FTLD with FUS pathology from amyotrophic lateral sclerosis with FUS mutations. Brain 134:2595–2609 21856723 10.1093/brain/awr201 PMC3170539

[CR233] Nguyen TB, Miramontes R, Chillon-Marinas C, Maimon R, Vazquez-Sanchez S, Lau AL, McClure NR, England WE, Singha M, Stocksdale JT et al (2023) Aberrant splicing in Huntington’s disease via disrupted TDP-43 activity accompanied by altered m6A RNA modification. Preprint at https://www.biorxiv.org/content/biorxiv/early/2023/11/02/2023.10.31.565004.full.pdf 10.1038/s41593-024-01850-w PMC11802453 39762660

[CR234] Nikom D, Zheng S (2023) Alternative splicing in neurodegenerative disease and the promise of RNA therapies. Nat Rev Neurosci 24:457–473 37336982 10.1038/s41583-023-00717-6

[CR235] Nishitoh H, Kadowaki H, Nagai A, Maruyama T, Yokota T, Fukutomi H, Noguchi T, Matsuzawa A, Takeda K, Ichijo H (2008) ALS-linked mutant SOD1 induces ER stress- and ASK1-dependent motor neuron death by targeting Derlin-1. Genes Dev 22:1451–1464 18519638 10.1101/gad.1640108 PMC2418582

[CR236] Nurk S, Koren S, Rhie A, Rautiainen M, Bzikadze AV, Mikheenko A, Vollger MR, Altemose N, Uralsky L, Gershman A et al (2022) The complete sequence of a human genome. Science 376:44–53 35357919 10.1126/science.abj6987 PMC9186530

[CR237] Nussbacher JK, Tabet R, Yeo GW, Lagier-Tourenne C (2019) Disruption of RNA metabolism in neurological diseases and emerging therapeutic interventions. Neuron 102:294–320 30998900 10.1016/j.neuron.2019.03.014 PMC6545120

[CR238] Olanow CW, Tatton WG (1999) Etiology and pathogenesis of Parkinson’s disease. Annu Rev Neurosci 22:123–144 10202534 10.1146/annurev.neuro.22.1.123

[CR239] Onofrj M, Ghilardi MF (1990) MPTP induced parkinsonian syndrome: long term follow-up and neurophysiological study. Ital J Neurol Sci 11:445–458 2272779 10.1007/BF02336564

[CR240] Ortega JA, Daley EL, Kour S, Samani M, Tellez L, Smith HS, Hall EA, Esengul YT, Tsai YH, Gendron TF et al (2020) Nucleocytoplasmic proteomic analysis uncovers eRF1 and nonsense-mediated decay as modifiers of ALS/FTD C9orf72 toxicity. Neuron 106:90–107.e113 32059759 10.1016/j.neuron.2020.01.020 PMC7272217

[CR241] Ortega JA, Sasselli IR, Boccitto M, Fleming AC, Fortuna TR, Li Y, Sato K, Clemons TD, McKenna ED, Nguyen TP et al (2023) CLIP-Seq analysis enables the design of protective ribosomal RNA bait oligonucleotides against C9ORF72 ALS/FTD poly-GR pathophysiology. Sci Adv 9:eadf7997 37948524 10.1126/sciadv.adf7997 PMC10637751

[CR242] Otte CG, Fortuna TR, Mann JR, Gleixner AM, Ramesh N, Pyles NJ, Pandey UB, Donnelly CJ (2020) Optogenetic TDP-43 nucleation induces persistent insoluble species and progressive motor dysfunction in vivo. Neurobiol Dis 146:105078 32927062 10.1016/j.nbd.2020.105078 PMC9040199

[CR243] Park J, Lee J, Kim JH, Lee J, Park H, Lim C (2021) ZNF598 co-translationally titrates poly(GR) protein implicated in the pathogenesis of C9ORF72-associated ALS/FTD. Nucleic Acids Res 49:11294–11311 34551427 10.1093/nar/gkab834 PMC8565315

[CR244] Pascual-Morena C, Cavero-Redondo I, Alvarez-Bueno C, Mesas AE, Pozuelo-Carrascosa D, Martinez-Vizcaino V (2020) Restorative treatments of dystrophin expression in Duchenne muscular dystrophy: a systematic review. Ann Clin Transl Neurol 7:1738–1752 33325654 10.1002/acn3.51149 PMC7480922

[CR245] Passmore LA, Coller J (2022) Roles of mRNA poly(A) tails in regulation of eukaryotic gene expression. Nat Rev Mol Cell Biol 23:93–106 34594027 10.1038/s41580-021-00417-y PMC7614307

[CR246] Patel A, Lee HO, Jawerth L, Maharana S, Jahnel M, Hein MY, Stoynov S, Mahamid J, Saha S, Franzmann TM et al (2015) A liquid-to-solid phase transition of the ALS protein FUS accelerated by disease mutation. Cell 162:1066–1077 26317470 10.1016/j.cell.2015.07.047

[CR247] Patel R, Brophy C, Hickling M, Neve J, Furger A (2019) Alternative cleavage and polyadenylation of genes associated with protein turnover and mitochondrial function are deregulated in Parkinson’s, Alzheimer’s and ALS disease. BMC Med Genomics 12:60 31072331 10.1186/s12920-019-0509-4 PMC6507032

[CR248] Paulson H (2018) Repeat expansion diseases. Handb Clin Neurol 147:105–123 29325606 10.1016/B978-0-444-63233-3.00009-9 PMC6485936

[CR249] Perez-Arancibia R, Cisternas-Olmedo M, Sepulveda D, Troncoso-Escudero P, Vidal RL (2022) Small molecules to perform big roles: the search for Parkinson’s and Huntington’s disease therapeutics. Front Neurosci 16:1084493 36699535 10.3389/fnins.2022.1084493 PMC9868863

[CR250] Pickrell AM, Youle RJ (2015) The roles of PINK1, parkin, and mitochondrial fidelity in Parkinson’s disease. Neuron 85:257–273 25611507 10.1016/j.neuron.2014.12.007 PMC4764997

[CR251] Polymenidou M, Lagier-Tourenne C, Hutt KR, Huelga SC, Moran J, Liang TY, Ling SC, Sun E, Wancewicz E, Mazur C et al (2011) Long pre-mRNA depletion and RNA missplicing contribute to neuronal vulnerability from loss of TDP-43. Nat Neurosci 14:459–468 21358643 10.1038/nn.2779 PMC3094729

[CR252] Prostko CR, Dholakia JN, Brostrom MA, Brostrom CO (1995) Activation of the double-stranded RNA-regulated protein kinase by depletion of endoplasmic reticular calcium stores. J Biol Chem 270:6211–6215 7890757 10.1074/jbc.270.11.6211

[CR253] Prudencio M, Belzil VV, Batra R, Ross CA, Gendron TF, Pregent LJ, Murray ME, Overstreet KK, Piazza-Johnston AE, Desaro P et al (2015) Distinct brain transcriptome profiles in C9orf72-associated and sporadic ALS. Nat Neurosci 18:1175–1182 26192745 10.1038/nn.4065 PMC4830686

[CR254] Pun FW, Liu BHM, Long X, Leung HW, Leung GHD, Mewborne QT, Gao J, Shneyderman A, Ozerov IV, Wang J et al (2022) Identification of therapeutic targets for amyotrophic lateral sclerosis using PandaOmics—an AI-enabled biological target discovery platform. Front Aging Neurosci 14:914017 35837482 10.3389/fnagi.2022.914017 PMC9273868

[CR255] Qamar S, Wang G, Randle SJ, Ruggeri FS, Varela JA, Lin JQ, Phillips EC, Miyashita A, Williams D, Strohl F et al (2018) FUS phase separation is modulated by a molecular chaperone and methylation of arginine cation-pi interactions. Cell 173:720–734.e715 29677515 10.1016/j.cell.2018.03.056 PMC5927716

[CR256] Radwan M, Ang CS, Ormsby AR, Cox D, Daly JC, Reid GE, Hatters DM (2020) Arginine in C9ORF72 dipolypeptides mediates promiscuous proteome binding and multiple modes of toxicity. Mol Cell Proteom 19:640–654 10.1074/mcp.RA119.001888 PMC7124463 32086375

[CR257] Raju GSR, Pavitra E, Bandaru SS, Varaprasad GL, Nagaraju GP, Malla RR, Huh YS, Han YK (2023) HOTAIR: a potential metastatic, drug-resistant and prognostic regulator of breast cancer. Mol Cancer 22:65 36997931 10.1186/s12943-023-01765-3 PMC10061914

[CR258] Rigo F, Hua Y, Krainer AR, Bennett CF (2012) Antisense-based therapy for the treatment of spinal muscular atrophy. J Cell Biol 199:21–25 23027901 10.1083/jcb.201207087 PMC3461520

[CR259] Roberts TC, Langer R, Wood MJA (2020) Advances in oligonucleotide drug delivery. Nat Rev Drug Discov 19:673–694 32782413 10.1038/s41573-020-0075-7 PMC7419031

[CR260] Rossi D, Volanti P, Brambilla L, Colletti T, Spataro R, La Bella V (2018) CSF neurofilament proteins as diagnostic and prognostic biomarkers for amyotrophic lateral sclerosis. J Neurol 265:510–521 29322259 10.1007/s00415-017-8730-6

[CR261] Rot G, Wang Z, Huppertz I, Modic M, Lence T, Hallegger M, Haberman N, Curk T, von Mering C, Ule J (2017) High-resolution RNA maps suggest common principles of splicing and polyadenylation regulation by TDP-43. Cell Rep 19:1056–1067 28467899 10.1016/j.celrep.2017.04.028 PMC5437728

[CR262] Rothstein JD, Baskerville V, Rapuri S, Mehlhop E, Jafar-Nejad P, Rigo F, Bennett F, Mizielinska S, Isaacs A, Coyne AN (2023) G(2)C(4) targeting antisense oligonucleotides potently mitigate TDP-43 dysfunction in human C9orf72 ALS/FTD induced pluripotent stem cell derived neurons. Acta Neuropathol 147:1 38019311 10.1007/s00401-023-02652-3 PMC10840905

[CR263] Roundtree IA, Evans ME, Pan T, He C (2017) Dynamic RNA modifications in gene expression regulation. Cell 169:1187–1200 28622506 10.1016/j.cell.2017.05.045 PMC5657247

[CR264] Sakuma S, D’Angelo MA (2017) The roles of the nuclear pore complex in cellular dysfunction, aging and disease. Semin Cell Dev Biol 68:72–84 28506892 10.1016/j.semcdb.2017.05.006 PMC5568450

[CR265] San Juan IG, Nash LA, Smith KS, Leyton-Jaimes MF, Qian M, Klim JR, Limone F, Dorr AB, Couto A, Pintacuda G et al (2022) Loss of mouse Stmn2 function causes motor neuropathy. Neuron 110:4031 36480942 10.1016/j.neuron.2022.11.003

[CR266] Sauvageau M, Goff LA, Lodato S, Bonev B, Groff AF, Gerhardinger C, Sanchez-Gomez DB, Hacisuleyman E, Li E, Spence M et al (2013) Multiple knockout mouse models reveal lincRNAs are required for life and brain development. eLife 2:e01749 24381249 10.7554/eLife.01749 PMC3874104

[CR267] Savas JN, Toyama BH, Xu T, Yates 3rd JR, Hetzer MW (2012) Extremely long-lived nuclear pore proteins in the rat brain. Science 335:942 22300851 10.1126/science.1217421 PMC3296478

[CR268] Saxena S, Cabuy E, Caroni P (2009) A role for motoneuron subtype-selective ER stress in disease manifestations of FALS mice. Nat Neurosci 12:627–636 19330001 10.1038/nn.2297

[CR269] Schoch KM, DeVos SL, Miller RL, Chun SJ, Norrbom M, Wozniak DF, Dawson HN, Bennett CF, Rigo F, Miller TM (2016) Increased 4R-Tau induces pathological changes in a human-Tau mouse model. Neuron 90:941–947 27210553 10.1016/j.neuron.2016.04.042 PMC5040069

[CR270] Schwartz JL, Jones KL, Yeo GW (2021) Repeat RNA expansion disorders of the nervous system: post-transcriptional mechanisms and therapeutic strategies. Crit Rev Biochem Mol Biol 56:31–53 33172304 10.1080/10409238.2020.1841726 PMC8192115

[CR271] Schwartz S, Bernstein DA, Mumbach MR, Jovanovic M, Herbst RH, Leon-Ricardo BX, Engreitz JM, Guttman M, Satija R, Lander ES et al (2014) Transcriptome-wide mapping reveals widespread dynamic-regulated pseudouridylation of ncRNA and mRNA. Cell 159:148–162 25219674 10.1016/j.cell.2014.08.028 PMC4180118

[CR272] Scoles DR, Meera P, Schneider MD, Paul S, Dansithong W, Figueroa KP, Hung G, Rigo F, Bennett CF, Otis TS et al (2017) Antisense oligonucleotide therapy for spinocerebellar ataxia type 2. Nature 544:362–366 28405024 10.1038/nature22044 PMC6625650

[CR273] Seddighi S, Qi YA, Brown AL, Wilkins OG, Bereda C, Belair C, Zhang YJ, Prudencio M, Keuss MJ, Khandeshi A et al (2024) Mis-spliced transcripts generate de novo proteins in TDP-43-related ALS/FTD. Sci Transl Med 16:eadg7162 38277467 10.1126/scitranslmed.adg7162 PMC11325748

[CR274] Segev Y, Barrera I, Ounallah-Saad H, Wibrand K, Sporild I, Livne A, Rosenberg T, David O, Mints M, Bramham CR et al (2015) PKR inhibition rescues memory deficit and ATF4 overexpression in ApoE epsilon4 human replacement mice. J Neurosci 35:12986–12993 26400930 10.1523/JNEUROSCI.5241-14.2015 PMC6605432

[CR275] Setten RL, Rossi JJ, Han SP (2019) The current state and future directions of RNAi-based therapeutics. Nat Rev Drug Discov 18:421–446 30846871 10.1038/s41573-019-0017-4

[CR276] Sheffield LG, Miskiewicz HB, Tannenbaum LB, Mirra SS (2006) Nuclear pore complex proteins in Alzheimer disease. J Neuropathol Exp Neurol 65:45–54 16410748 10.1097/01.jnen.0000195939.40410.08

[CR277] Shi KY, Mori E, Nizami ZF, Lin Y, Kato M, Xiang S, Wu LC, Ding M, Yu Y, Gall JG et al (2017) Toxic PR(n) poly-dipeptides encoded by the C9orf72 repeat expansion block nuclear import and export. Proc Natl Acad Sci USA 114:E1111–E1117 28069952 10.1073/pnas.1620293114 PMC5320981

[CR278] Shimobayashi SF, Ronceray P, Sanders DW, Haataja MP, Brangwynne CP (2021) Nucleation landscape of biomolecular condensates. Nature 599:503–506 34552246 10.1038/s41586-021-03905-5

[CR279] Shirley M (2021) Casimersen: first approval. Drugs 81:875–879 33861387 10.1007/s40265-021-01512-2

[CR280] Simchovitz A, Hanan M, Niederhoffer N, Madrer N, Yayon N, Bennett ER, Greenberg DS, Kadener S, Soreq H (2019) NEAT1 is overexpressed in Parkinson’s disease substantia nigra and confers drug-inducible neuroprotection from oxidative stress. FASEB J 33:11223–11234 31311324 10.1096/fj.201900830R PMC6766647

[CR281] Sloan KE, Warda AS, Sharma S, Entian KD, Lafontaine DLJ, Bohnsack MT (2017) Tuning the ribosome: the influence of rRNA modification on eukaryotic ribosome biogenesis and function. RNA Biol 14:1138–1152 27911188 10.1080/15476286.2016.1259781 PMC5699541

[CR282] Spector DL, Lamond AI (2011) Nuclear speckles. Cold Spring Harb Perspect Biol 3:a000646 20926517 10.1101/cshperspect.a000646 PMC3039535

[CR283] Staffaroni AM, Quintana M, Wendelberger B, Heuer HW, Russell LL, Cobigo Y, Wolf A, Goh SM, Petrucelli L, Gendron TF et al (2022) Temporal order of clinical and biomarker changes in familial frontotemporal dementia. Nat Med 28:2194–2206 36138153 10.1038/s41591-022-01942-9 PMC9951811

[CR284] Statello L, Guo CJ, Chen LL, Huarte M (2021) Gene regulation by long non-coding RNAs and its biological functions. Nat Rev Mol Cell Biol 22:96–118 33353982 10.1038/s41580-020-00315-9 PMC7754182

[CR285] Storkebaum E, Rosenblum K, Sonenberg N (2023) Messenger RNA translation defects in neurodegenerative diseases. New Engl J Med 388:1015–1030 36920757 10.1056/NEJMra2215795

[CR286] Sulston JE, Brenner S (1974) The DNA of *Caenorhabditis elegans*. Genetics 77:95–104 4858229 10.1093/genetics/77.1.95 PMC1213121

[CR287] Sun M, Bell W, LaClair KD, Ling JP, Han H, Kageyama Y, Pletnikova O, Troncoso JC, Wong PC, Chen LL (2017) Cryptic exon incorporation occurs in Alzheimer’s brain lacking TDP-43 inclusion but exhibiting nuclear clearance of TDP-43. Acta Neuropathol 133:923–931 28332094 10.1007/s00401-017-1701-2 PMC5444385

[CR288] Sun Q, Zhang Y, Wang S, Yang F, Cai H, Xing Y, Zhou L, Chen S, Wang Y (2022) LncRNA HOTAIR promotes alpha-synuclein aggregation and apoptosis of SH-SY5Y cells by regulating miR-221-3p in Parkinson’s disease. Exp Cell Res 417:113132 35398161 10.1016/j.yexcr.2022.113132

[CR289] Sun S, Ling SC, Qiu J, Albuquerque CP, Zhou Y, Tokunaga S, Li H, Qiu H, Bui A, Yeo GW et al (2015a) ALS-causative mutations in FUS/TLS confer gain and loss of function by altered association with SMN and U1-snRNP. Nat Commun 6:6171 25625564 10.1038/ncomms7171 PMC4338613

[CR290] Sun S, Sun Y, Ling SC, Ferraiuolo L, McAlonis-Downes M, Zou Y, Drenner K, Wang Y, Ditsworth D, Tokunaga S et al (2015b) Translational profiling identifies a cascade of damage initiated in motor neurons and spreading to glia in mutant SOD1-mediated ALS. Proc Natl Acad Sci USA 112:E6993–E7002 26621731 10.1073/pnas.1520639112 PMC4687558

[CR291] Sun Y, Dai H, Dai X, Yin J, Cui Y, Liu X, Gonzalez G, Yuan J, Tang F, Wang N et al (2023) m(1)A in CAG repeat RNA binds to TDP-43 and induces neurodegeneration. Nature 623:580–587 37938769 10.1038/s41586-023-06701-5 PMC10651481

[CR292] Sun Y, Eshov A, Zhou J, Isiktas AU, Guo JU (2020) C9orf72 arginine-rich dipeptide repeats inhibit UPF1-mediated RNA decay via translational repression. Nat Commun 11:3354 32620797 10.1038/s41467-020-17129-0 PMC7335171

[CR293] Takanashi K, Yamaguchi A (2014) Aggregation of ALS-linked FUS mutant sequesters RNA binding proteins and impairs RNA granules formation. Biochem Biophys Res Commun 452:600–607 25173930 10.1016/j.bbrc.2014.08.115

[CR294] Takuma H, Kwak S, Yoshizawa T, Kanazawa I (1999) Reduction of GluR2 RNA editing, a molecular change that increases calcium influx through AMPA receptors, selective in the spinal ventral gray of patients with amyotrophic lateral sclerosis. Ann Neurol 46:806–815 10589532 10.1002/1531-8249(199912)46:6<806::aid-ana2>3.0.co;2-s

[CR295] Tan CL, Plotkin JL, Veno MT, von Schimmelmann M, Feinberg P, Mann S, Handler A, Kjems J, Surmeier DJ, O’Carroll D et al (2013) MicroRNA-128 governs neuronal excitability and motor behavior in mice. Science 342:1254–1258 24311694 10.1126/science.1244193 PMC3932786

[CR296] Tank EM, Figueroa-Romero C, Hinder LM, Bedi K, Archbold HC, Li X, Weskamp K, Safren N, Paez-Colasante X, Pacut C et al (2018) Abnormal RNA stability in amyotrophic lateral sclerosis. Nat Commun 9:2845 30030424 10.1038/s41467-018-05049-z PMC6054632

[CR297] Tetter S, Arseni D, Murzin AG, Buhidma Y, Peak-Chew SY, Garringer HJ, Newell KL, Vidal R, Apostolova LG, Lashley T et al (2024) TAF15 amyloid filaments in frontotemporal lobar degeneration. Nature 625:345–351 38057661 10.1038/s41586-023-06801-2 PMC10781619

[CR298] Tian B, Manley JL (2017) Alternative polyadenylation of mRNA precursors. Nat Rev Mol Cell Biol 18:18–30 27677860 10.1038/nrm.2016.116 PMC5483950

[CR299] Tible M, Mouton Liger F, Schmitt J, Giralt A, Farid K, Thomasseau S, Gourmaud S, Paquet C, Rondi Reig L, Meurs E et al (2019) PKR knockout in the 5xFAD model of Alzheimer’s disease reveals beneficial effects on spatial memory and brain lesions. Aging Cell 18:e12887 30821420 10.1111/acel.12887 PMC6516179

[CR300] Tollervey JR, Curk T, Rogelj B, Briese M, Cereda M, Kayikci M, Konig J, Hortobagyi T, Nishimura AL, Zupunski V et al (2011) Characterizing the RNA targets and position-dependent splicing regulation by TDP-43. Nat Neurosci 14:452–458 21358640 10.1038/nn.2778 PMC3108889

[CR301] Tran H, Moazami MP, Yang H, McKenna-Yasek D, Douthwright CL, Pinto C, Metterville J, Shin M, Sanil N, Dooley C et al (2022) Suppression of mutant C9orf72 expression by a potent mixed backbone antisense oligonucleotide. Nat Med 28:117–124 34949835 10.1038/s41591-021-01557-6 PMC8861976

[CR302] Tsai YL, Mu YC, Manley JL (2022) Nuclear RNA transcript levels modulate nucleocytoplasmic distribution of ALS/FTD-associated protein FUS. Sci Rep 12:8180 35581240 10.1038/s41598-022-12098-4 PMC9114323

[CR303] van den Berg LH, Rothstein JD, Shaw PJ, Babu S, Benatar M, Bucelli RC, Genge A, Glass JD, Hardiman O, Libri V et al (2024) Safety, tolerability, and pharmacokinetics of antisense oligonucleotide BIIB078 in adults with C9orf72-associated amyotrophic lateral sclerosis: a phase 1, randomised, double blinded, placebo-controlled, multiple ascending dose study. Lancet Neurol 23:901–912 39059407 10.1016/S1474-4422(24)00216-3

[CR304] van Hoof A, Frischmeyer PA, Dietz HC, Parker R (2002) Exosome-mediated recognition and degradation of mRNAs lacking a termination codon. Science 295:2262–2264 11910110 10.1126/science.1067272

[CR305] Vance C, Rogelj B, Hortobagyi T, De Vos KJ, Nishimura AL, Sreedharan J, Hu X, Smith B, Ruddy D, Wright P et al (2009) Mutations in FUS, an RNA processing protein, cause familial amyotrophic lateral sclerosis type 6. Science 323:1208–1211 19251628 10.1126/science.1165942 PMC4516382

[CR306] Vatsavayai SC, Yoon SJ, Gardner RC, Gendron TF, Vargas JN, Trujillo A, Pribadi M, Phillips JJ, Gaus SE, Hixson JD et al (2016) Timing and significance of pathological features in C9orf72 expansion-associated frontotemporal dementia. Brain 139:3202–3216 27797809 10.1093/brain/aww250 PMC5790143

[CR307] Verde F, Steinacker P, Weishaupt JH, Kassubek J, Oeckl P, Halbgebauer S, Tumani H, von Arnim CAF, Dorst J, Feneberg E et al (2019) Neurofilament light chain in serum for the diagnosis of amyotrophic lateral sclerosis. J Neurol Neurosurg Psychiatry 90:157–164 30309882 10.1136/jnnp-2018-318704

[CR308] Wang C, Duan Y, Duan G, Wang Q, Zhang K, Deng X, Qian B, Gu J, Ma Z, Zhang S et al (2020) Stress induces dynamic, cytotoxicity-antagonizing TDP-43 nuclear bodies via paraspeckle LncRNA NEAT1-mediated liquid-liquid phase separation. Mol Cell 79:443–458.e447 32649883 10.1016/j.molcel.2020.06.019

[CR309] Wang CX, Cui GS, Liu X, Xu K, Wang M, Zhang XX, Jiang LY, Li A, Yang Y, Lai WY et al (2018) METTL3-mediated m^6^A modification is required for cerebellar development. PLoS Biol 16:e2004880 29879109 10.1371/journal.pbio.2004880 PMC6021109

[CR310] Wang ET, Sandberg R, Luo S, Khrebtukova I, Zhang L, Mayr C, Kingsmore SF, Schroth GP, Burge CB (2008) Alternative isoform regulation in human tissue transcriptomes. Nature 456:470–476 18978772 10.1038/nature07509 PMC2593745

[CR311] Wang S, Sun S (2023) Translation dysregulation in neurodegenerative diseases: a focus on ALS. Mol Neurodegener 18:58 37626421 10.1186/s13024-023-00642-3 PMC10464328

[CR312] Wang S, Zhang X, Guo Y, Rong H, Liu T (2017) The long noncoding RNA HOTAIR promotes Parkinson’s disease by upregulating LRRK2 expression. Oncotarget 8:24449–24456 28445933 10.18632/oncotarget.15511 PMC5421861

[CR313] Watanabe S, Oiwa K, Murata Y, Komine O, Sobue A, Endo F, Takahashi E, Yamanaka K (2020) ALS-linked TDP-43(M337V) knock-in mice exhibit splicing deregulation without neurodegeneration. Mol Brain 13:8 31959210 10.1186/s13041-020-0550-4 PMC6971932

[CR314] Weskamp K, Barmada SJ (2018) RNA degradation in neurodegenerative disease. Adv Neurobiol 20:103–142 29916018 10.1007/978-3-319-89689-2_5 PMC6498147

[CR315] Westergard T, McAvoy K, Russell K, Wen X, Pang Y, Morris B, Pasinelli P, Trotti D, Haeusler A (2019) Repeat-associated non-AUG translation in C9orf72-ALS/FTD is driven by neuronal excitation and stress. EMBO Mol Med 11:e9423 30617154 10.15252/emmm.201809423 PMC6365928

[CR316] Westholm JO, Miura P, Olson S, Shenker S, Joseph B, Sanfilippo P, Celniker SE, Graveley BR, Lai EC (2014) Genome-wide analysis of Drosophila circular RNAs reveals their structural and sequence properties and age-dependent neural accumulation. Cell Rep 9:1966–1980 25544350 10.1016/j.celrep.2014.10.062 PMC4279448

[CR317] Wingo TS, Liu Y, Gerasimov ES, Vattathil SM, Wynne ME, Liu J, Lori A, Faundez V, Bennett DA, Seyfried NT et al (2022) Shared mechanisms across the major psychiatric and neurodegenerative diseases. Nat Commun 13:4314 35882878 10.1038/s41467-022-31873-5 PMC9325708

[CR318] Wolozin B (2012) Regulated protein aggregation: stress granules and neurodegeneration. Mol Neurodegener 7:56 23164372 10.1186/1750-1326-7-56 PMC3519755

[CR319] Wright AL, Konen LM, Mockett BG, Morris GP, Singh A, Burbano LE, Milham L, Hoang M, Zinn R, Chesworth R et al (2023) The Q/R editing site of AMPA receptor GluA2 subunit acts as an epigenetic switch regulating dendritic spines, neurodegeneration and cognitive deficits in Alzheimer’s disease. Mol Neurodegener 18:65 37759260 10.1186/s13024-023-00632-5 PMC10537207

[CR320] Wright SE, Rodriguez CM, Monroe J, Xing J, Krans A, Flores BN, Barsur V, Ivanova MI, Koutmou KS, Barmada SJ et al (2022) CGG repeats trigger translational frameshifts that generate aggregation-prone chimeric proteins. Nucleic Acids Res 50:8674–8689 35904811 10.1093/nar/gkac626 PMC9410890

[CR321] Wu R, Ye Y, Dong D, Zhang Z, Wang S, Li Y, Wright N, Redding-Ochoa J, Chang K, Xu S et al (2024) Disruption of nuclear speckle integrity dysregulates RNA splicing in C9ORF72-FTD/ALS. Neuron 112:3434–3451 10.1016/j.neuron.2024.07.025 PMC11502262 39181135

[CR322] Xia D, Sui R, Zhang Z (2019) Administration of resveratrol improved Parkinson’s disease-like phenotype by suppressing apoptosis of neurons via modulating the MALAT1/miR-129/SNCA signaling pathway. J Cell Biochem 120:4942–4951 30260025 10.1002/jcb.27769

[CR323] Xu W, Bao P, Jiang X, Wang H, Qin M, Wang R, Wang T, Yang Y, Lorenzini I, Liao L et al (2019) Reactivation of nonsense-mediated mRNA decay protects against C9orf72 dipeptide-repeat neurotoxicity. Brain 142:1349–1364 30938419 10.1093/brain/awz070 PMC6487333

[CR324] Xu Z, Poidevin M, Li X, Li Y, Shu L, Nelson DL, Li H, Hales CM, Gearing M, Wingo TS et al (2013) Expanded GGGGCC repeat RNA associated with amyotrophic lateral sclerosis and frontotemporal dementia causes neurodegeneration. Proc Natl Acad Sci USA 110:7778–7783 23553836 10.1073/pnas.1219643110 PMC3651485

[CR325] Yamashita T, Hideyama T, Hachiga K, Teramoto S, Takano J, Iwata N, Saido TC, Kwak S (2012) A role for calpain-dependent cleavage of TDP-43 in amyotrophic lateral sclerosis pathology. Nat Commun 3:1307 23250437 10.1038/ncomms2303

[CR326] Yamazaki T, Chen S, Yu Y, Yan B, Haertlein TC, Carrasco MA, Tapia JC, Zhai B, Das R, Lalancette-Hebert M et al (2012) FUS-SMN protein interactions link the motor neuron diseases ALS and SMA. Cell Rep 2:799–806 23022481 10.1016/j.celrep.2012.08.025 PMC3483417

[CR327] Yang S, Lim KH, Kim SH, Joo JY (2021) Molecular landscape of long noncoding RNAs in brain disorders. Mol Psychiatry 26:1060–1074 33173194 10.1038/s41380-020-00947-5

[CR328] Yang Y, Guo L, Chen L, Gong B, Jia D, Sun Q (2023) Nuclear transport proteins: structure, function, and disease relevance. Signal Transduct Target Ther 8:425 37945593 10.1038/s41392-023-01649-4 PMC10636164

[CR329] Yin S, Lopez-Gonzalez R, Kunz RC, Gangopadhyay J, Borufka C, Gygi SP, Gao FB, Reed R (2017) Evidence that C9ORF72 dipeptide repeat proteins associate with U2 snRNP to cause mis-splicing in ALS/FTD patients. Cell Rep 19:2244–2256 28614712 10.1016/j.celrep.2017.05.056 PMC5653973

[CR330] Yu H, Lu S, Gasior K, Singh D, Vazquez-Sanchez S, Tapia O, Toprani D, Beccari MS, Yates 3rd JR, Da Cruz S et al (2021) HSP70 chaperones RNA-free TDP-43 into anisotropic intranuclear liquid spherical shells. Science 371:eabb4309 33335017 10.1126/science.abb4309 PMC8286096

[CR331] Zaepfel BL, Zhang Z, Maulding K, Coyne AN, Cheng W, Hayes LR, Lloyd TE, Sun S, Rothstein JD (2021) UPF1 reduces C9orf72 HRE-induced neurotoxicity in the absence of nonsense-mediated decay dysfunction. Cell Rep 34:108925 33789100 10.1016/j.celrep.2021.108925 PMC8063722

[CR332] Zeng Y, Lovchykova A, Akiyama T, Liu C, Guo C, Jawahar VM, Sianto O, Calliari A, Prudencio M, Dickson DW et al (2024) TDP-43 nuclear loss in FTD/ALS causes widespread alternative polyadenylation changes. Preprint at https://www.biorxiv.org/content/10.1101/2024.01.22.575730v1 10.1038/s41593-025-02049-3 PMC12586192 41120750

[CR333] Zetsche B, Volz SE, Zhang F (2015) A split-Cas9 architecture for inducible genome editing and transcription modulation. Nat Biotechnol 33:139–142 25643054 10.1038/nbt.3149 PMC4503468

[CR334] Zhang K, Daigle JG, Cunningham KM, Coyne AN, Ruan K, Grima JC, Bowen KE, Wadhwa H, Yang P, Rigo F et al (2018a) Stress granule assembly disrupts nucleocytoplasmic transport. Cell 173:958–971.e917 29628143 10.1016/j.cell.2018.03.025 PMC6083872

[CR335] Zhang QS, Wang ZH, Zhang JL, Duan YL, Li GF, Zheng DL (2016a) Beta-asarone protects against MPTP-induced Parkinson’s disease via regulating long non-coding RNA MALAT1 and inhibiting alpha-synuclein protein expression. Biomed Pharmacother 83:153–159 27470562 10.1016/j.biopha.2016.06.017

[CR336] Zhang S, Cooper-Knock J, Weimer AK, Shi M, Moll T, Marshall JNG, Harvey C, Nezhad HG, Franklin J, Souza CDS et al (2022) Genome-wide identification of the genetic basis of amyotrophic lateral sclerosis. Neuron 110:992–1008.e1011 35045337 10.1016/j.neuron.2021.12.019 PMC9017397

[CR337] Zhang Y, Xue W, Li X, Zhang J, Chen S, Zhang JL, Yang L, Chen LL (2016b) The biogenesis of nascent circular RNAs. Cell Rep 15:611–624 27068474 10.1016/j.celrep.2016.03.058

[CR338] Zhang YJ, Gendron TF, Ebbert MTW, O’Raw AD, Yue M, Jansen-West K, Zhang X, Prudencio M, Chew J, Cook CN et al (2018b) Poly(GR) impairs protein translation and stress granule dynamics in C9orf72-associated frontotemporal dementia and amyotrophic lateral sclerosis. Nat Med 24:1136–1142 29942091 10.1038/s41591-018-0071-1 PMC6520050

[CR339] Zhang YJ, Jansen-West K, Xu YF, Gendron TF, Bieniek KF, Lin WL, Sasaguri H, Caulfield T, Hubbard J, Daughrity L et al (2014) Aggregation-prone c9FTD/ALS poly(GA) RAN-translated proteins cause neurotoxicity by inducing ER stress. Acta Neuropathol 128:505–524 25173361 10.1007/s00401-014-1336-5 PMC4159567

[CR340] Zhang Z, Lotti F, Dittmar K, Younis I, Wan L, Kasim M, Dreyfuss G (2008) SMN deficiency causes tissue-specific perturbations in the repertoire of snRNAs and widespread defects in splicing. Cell 133:585–600 18485868 10.1016/j.cell.2008.03.031 PMC2446403

[CR341] Zhao F, Xu Y, Gao S, Qin L, Austria Q, Siedlak SL, Pajdzik K, Dai Q, He C, Wang W et al (2021) METTL3-dependent RNA m^6^A dysregulation contributes to neurodegeneration in Alzheimer’s disease through aberrant cell cycle events. Mol Neurodegener 16:70 34593014 10.1186/s13024-021-00484-x PMC8482683

[CR342] Zhu Y, Zhu L, Wang X, Jin H (2022) RNA-based therapeutics: an overview and prospectus. Cell Death Dis 13:644 35871216 10.1038/s41419-022-05075-2 PMC9308039

[CR343] Zu T, Guo S, Bardhi O, Ryskamp DA, Li J, Khoramian Tusi S, Engelbrecht A, Klippel K, Chakrabarty P, Nguyen L et al (2020) Metformin inhibits RAN translation through PKR pathway and mitigates disease in C9orf72 ALS/FTD mice. Proc Natl Acad Sci USA 117:18591–18599 32690681 10.1073/pnas.2005748117 PMC7414156

